# Understanding Public Attitudes and Willingness to Share Commercial Data for Health Research: Survey Study in the United Kingdom

**DOI:** 10.2196/40814

**Published:** 2023-03-23

**Authors:** Yasemin Hirst, Sandro T Stoffel, Hannah R Brewer, Lada Timotijevic, Monique M Raats, James M Flanagan

**Affiliations:** 1 Lancaster Medical School Lancaster University Lancaster United Kingdom; 2 Department of Behavioural Science and Health University College London London United Kingdom; 3 Department of Surgery and Cancer Imperial College London London United Kingdom; 4 Institute of Pharmaceutical Medicine University of Basel Basel Switzerland; 5 School of Psychology University of Surrey Guildford United Kingdom; 6 Surrey Institute for People-Centred Artificial Intelligence University of Surrey Guildford United Kingdom; 7 Institute for Sustainability University of Surrey Guildford United Kingdom

**Keywords:** commercial data, data sharing, participant recruitment, loyalty cards, sociodemographic factors, data donation, data, health, public, acceptability, digital, mobile phone

## Abstract

**Background:**

Health research using commercial data is increasing. The evidence on public acceptability and sociodemographic characteristics of individuals willing to share commercial data for health research is scarce.

**Objective:**

This survey study investigates the willingness to share commercial data for health research in the United Kingdom with 3 different organizations (government, private, and academic institutions), 5 different data types (internet, shopping, wearable devices, smartphones, and social media), and 10 different invitation methods to recruit participants for research studies with a focus on sociodemographic characteristics and psychological predictors.

**Methods:**

We conducted a web-based survey using quota sampling based on age distribution in the United Kingdom in July 2020 (N=1534). Chi-squared tests tested differences by sociodemographic characteristics, and adjusted ordered logistic regressions tested associations with trust, perceived importance of privacy, worry about data misuse and perceived risks, and perceived benefits of data sharing. The results are shown as percentages, adjusted odds ratios, and 95% CIs.

**Results:**

Overall, 61.1% (937/1534) of participants were willing to share their data with the government and 61% (936/1534) of participants were willing to share their data with academic research institutions compared with 43.1% (661/1534) who were willing to share their data with private organizations. The willingness to share varied between specific types of data—51.8% (794/1534) for loyalty cards, 35.2% (540/1534) for internet search history, 32% (491/1534) for smartphone data, 31.8% (488/1534) for wearable device data, and 30.4% (467/1534) for social media data. Increasing age was consistently and negatively associated with all the outcomes. Trust was positively associated with willingness to share commercial data, whereas worry about data misuse and the perceived importance of privacy were negatively associated with willingness to share commercial data. The perceived risk of sharing data was positively associated with willingness to share when the participants considered all the specific data types but not with the organizations. The participants favored postal research invitations over digital research invitations.

**Conclusions:**

This UK-based survey study shows that willingness to share commercial data for health research varies; however, researchers should focus on effectively communicating their data practices to minimize concerns about data misuse and improve public trust in data science. The results of this study can be further used as a guide to consider methods to improve recruitment strategies in health-related research and to improve response rates and participant retention.

## Introduction

Health researchers are increasingly aiming to include accurate personal information collected outside of health care settings, including commercial data collected or processed for businesses relating to their customers (eg, internet searches, social media, loyalty cards, wearable devices, and mobile phone apps), to enhance our understanding of individuals’ health-related behaviors and health outcomes. With the rise of different data sources to track, monitor, and forecast disease and health outcomes, interest in carrying out research using individual commercial data has grown substantially [1-3]. However, much of this valuable research is often criticized for its representativeness and the low participation rates associated with public attitudes toward data sharing.

Evidence on the public acceptability of sharing health-related data is vast and suggests that improving the transparency of data collection and processing practices across institutions, creating trustworthy data ecosystems, and providing agency and data stewardship for data participants can improve the willingness to take part in research and share data [4-12]. The evidence for willingness to share data across different contexts varies depending on the type, purpose, and use of data. These often show that the public has some understanding of how data are being used and equally suggest that raising awareness about data practices does not increase willingness to share data [9]. A recent report highlights that further research is needed to improve public trust in light of the Cambridge Analytica scandal and other reported data misuse incidences [12].

Furthermore, with the implementation of the General Data Protection Regulation (GDPR) in 2018 [13], all individuals were given the right to carry out a subject access request from any organization that holds any information about them, thus allowing researchers to start analyzing new types of data sets with individual consent to understand behaviors such as diet [14], self-medication [15], and cancer risk [16] using purchase history recorded on loyalty cards; that is, an identity card issued by retailers to its customers to collect information on buyer behavior and generate reward schemes. However, a common limitation is the small sample size and biased population of individuals who are more willing to share their data [17]. A number of qualitative studies investigated willingness to share commercial data, specifically loyalty cards for health research, echoing the principal evidence shared across disciplines, as discussed earlier [15,18,19]. In contrast, for mobile and biosensor data sharing, there is a growing body of literature on the importance of understanding nonparticipation and willingness to share mobile phone apps and biosensor data [20-24]. A study that took place in England before the GDPR highlighted that, in the context of mobile data sharing, user behavior is also associated with willingness to share passive or actively collected mobile data [21]. Further experimental studies have highlighted the behavior of sharing mobile data, which requires capabilities from the users to fulfill the task and the characteristics of the individuals, framing of the request, emphasis on control over data, and assurances of privacy and confidentiality [22]. The implications of the willingness to share smartphone and sensor data with researchers are further understood in studies where the response rate for data sharing is less than 15%, and the representativeness of the population that shares the data is less than optimal [23]. This highlights the importance of understanding the characteristics of the population who are willing to share commercial data sets before data collection so that strategies can be developed to improve response rates and minimize bias.

Therefore, this study aims to investigate GDPR awareness and sociodemographic and psychological factors associated with the willingness to share commercial data for health research purposes after the implementation of the GDPR in the United Kingdom. Furthermore, it aimed to provide a summary of the public’s awareness of GDPR in 2020, 2 years after the GDPR and Data Protection Act 2018 were enacted in the United Kingdom. The GDPR has been kept in UK law as the UK GDPR [24]. For epidemiological research to advance using commercial data sets and effectively recruit participants, it is important to investigate the factors associated with the willingness to share commercial data for health research.

## Methods

### Setting and Design

A 10-minute web-based survey was conducted in the United Kingdom in August 2020 via Survey Monkey using Dynata International Limited. Nonprobability quota sampling was used for an adequate representation of different age groups in the United Kingdom, with the aim of recruiting 1500 participants to achieve a 1:10 participant-to-item ratio [25]. The distribution of the sample, respectively, based on age and sex distribution in the UK population was 18 to 29 years (20%), 30 to 39 years (17%), 40 to 49 years (18.5%), 50 to 59 years (15.5%), 60 to 69 years (14%), >70 years (15%), male (49.4%), and female (50.6%) [26].

### Ethics Approval

The project was reviewed by the University of College London Research Ethics Committee and received a favorable opinion (ref: 18095/001) and reported using the Strengthening the Reporting of Observational Studies in Epidemiology guidelines for cross-sectional research [27]. Information regarding ethics approval can be obtained directly from the University of College London Research Ethics Committee. The survey study only included anonymized data from the participants; therefore, the research team had no contact with the participants following their participation in the study. If individuals dropped out of the survey before its completion, this was considered a withdrawal from the study, and no data were included. Participants were paid a small monetary incentive through Dynata International Limited in line with their participant payment policies.

### Survey Measures

All measures with their item heritage are included in Multimedia Appendix 1. There were 3 primary outcome measures. These were the willingness to share commercial data for health research with different institutions (government, private, and academic), the willingness to share different types of commercial data (internet searches, social media, shopping data on loyalty cards, wearable devices, and mobile phone apps) with academic institutions, and the willingness to participate in health research based on different invitation sources. The rationale for these outcomes is as follows. In comparison to government and private organizations, which are often the primary data controllers for health, administrative, and commercial data, researchers at academic institutions often need to request access to data collected and controlled by government and private organizations. The differences between institutions were considered to understand the potential baseline response rate for potential research projects that aim to use commercial data in health research at academic institutions. The second primary outcome was then focused on willingness by data type and the extent to which this differentiates from the baseline willingness to share commercial data with academic institutions. The last outcome is included to consider how much willingness varied depending on the source of the invite to better understand the best ways to recruit participants who are more willing to share their data. All these outcomes were used to inform the communication strategies of a much larger academic project that aimed to recruit individual participants with informed consent requesting access to their commercial data, specifically loyalty card data from 2 UK-based high-street retailers, investigating self-care behaviors before ovarian cancer diagnosis [16].

The independent variables were included under four sections: (1) sociodemographic factors, including age, sex, marital status, education, ethnicity, and location in the United Kingdom; (2) the participants’ GDPR awareness; (3) psychological factors including trust in institutions, trust in data practices in academia, worry about data misuse, perceived risk in data sharing, perceived importance of privacy, and perceived benefit of data sharing; and (4) past experience taking part in health research and past experiences of data misuse.

### Statistical Analysis

We reported all measures and exclusions in this study and used complete case analysis without imputing missing data, as all questions were mandatory.

Factor analyses using principal component analysis (PCA) and reliability tests were carried out to ensure that the items included in various other studies measured the intended outcomes. Once the factors were identified, the scales were computed using total scores. A Cronbach alpha coefficient was calculated for each scale for internal consistency, and the interitem and interscale correlations were checked for internal consistency of items and scales. Each computed scale was reported using range, mean, SD, and Cronbach alpha coefficient.

Participant characteristics, self-reported GDPR awareness, and people’s awareness of personal data and GDPR law were reported using descriptive statistics. Some categorical items were recoded for ease of presentation and understanding of the differences in each category. Responses to items including “prefer not to say” and “other” were coded as missing because of the low cell count (<5 observations in each category) in sociodemographic items which would not have been coded negatively and subsequently excluded from the main analyses (40/1594, 2.5%). Primary outcome variables were recoded into “definitely yes” or “probably yes”=1 and “probably no” or “definitely no”=0 to compare 2 distinct intentions to share data for the comparison between sociodemographic characteristics of the participants [28]. Differences in the proportions of willingness to share commercial data were tested using chi-square statistics and reported in percentages. Ordered logistic regression was used to test for psychological factors associated with willingness to share commercial data for health research with different organizations and different types of data adjusted for the sociodemographic characteristics of the participants, previous research participation, and GDPR awareness. The variance explained by each model is included in Multimedia Appendix 1. Further ordered regression analyses were carried out for the different types of research invitations to identify whether there were sociodemographic factors associated with willingness to participate in health research (Multimedia Appendix 1). All results reported using adjusted odds ratios (aORs) and 95% CIs were reported using a *P* value of <.05.

## Results

### Factor Analysis Results

The 32 items measured in this study were subjected to PCA using SPSS (version 27; IBM Corp). Before performing PCA, the suitability for performing PCA was assessed. Inspection of the correlation matrix revealed the presence of many coefficients >0.3. The Kaiser-Meyer-Olkin value was 0.93, above the recommended value of 0.6, and the Bartlett Test of Sphericity reached statistical significance, supporting the factorability of the correlation matrix. The PCA revealed the presence of 5 components with eigenvalues exceeding 1, explaining 28.6%, 19.7%, 7.5%, 6.9%, and 5.3% variance (Multimedia Appendix 1). A total of 8 items were recoded, and 2 items were deleted, as they measured trust in 2 different organizations. On the basis of these results, 5 scales were computed. These are, namely, perceived importance of privacy (mean 8.46, SD 1.38; range 2-10; Cronbach α=.65), worry about data misuse (6 items; mean 21.28, SD 5.69; range 5-30; Cronbach α=.95), trust in data practices in academic institutions (9 items; mean 31.96, SD 7.14; range 5-45; Cronbach α=.95), perceived risk of data sharing for health research (3 items; mean 9.40, SD 2.73; range 3-15; Cronbach α=.88) and perceived benefits of data sharing (5 items; mean 17.76, SD 4.31; range 5-25; Cronbach α=.93). Factor correlations as separate scales suggest that the scales have weak to moderate correlations, indicating that they measure separate scales (Multimedia Appendix 1).

### Participant Characteristics

Out of the 1897 responses, 1534 participants gave their consent and completed the survey (Table 1). Approximately 49.1% (753/1534) of participants were male, 50.7% (777/1534) were female, and 0.2% (4/1534) indicated other. The age distribution of participants was consistent with the quota sample for the distribution of age in England. Most respondents self-identified with a White ethnic background (1325/1534, 86.4%), compared with only 12.9% (198/1534) who identified themselves with other ethnicities. Approximately 53.1% (814/1534) of the participants were married or had a legal partnership. Approximately 45.6% (699/1534) of participants had higher education (degree and above) qualifications, almost half of them (765/1534, 49.9%) had less than higher education qualifications, and only 4.5% (69/1534) did not have any educational qualification.

**Table 1:** Participant characteristics (N=1534).

Characteristics		Values, n (%)	Population composition of England and Wales based on 2011 Census (excludes Scotland and Northern Ireland) [21], %
**Sex**			
	Male	753 (49.1)	49.4
	Female	777 (50.7)	50.6
	Other	4 (0.2)	0
**Age (years)**			
	18-29	284 (18.5)	20
	30-39	255 (16.6)	17
	40-49	250 (16.3)	18.5
	50-59	266 (17.3)	15.5
	60-69	213 (13.9)	14
	≥70	266 (17.3)	15
**Ethnicity**			
	White British	1326 (87.0)	85.4
	Black	45 (3.0)	3.5
	Asian	111 (7.3)	7.1
	Mixed	32 (2.1)	2.3
	Other	10 (0.7)	1.0
**Marital status**			
	Single	492 (32.4)	34.5
	Married or legal partnership	814 (53.1)	50.8
	Widowed, divorced, or separated	212 (13.8)	14.6
**Educational level**			
	Higher education	697 (45.6)	27.1
	Higher education with qualification	762 (49.9)	49.9
	No qualification	68 (4.5)	23
**Location in the United Kingdom**			
	London	247 (16.2)	14.6
	East of England	149 (9.8)	10.4
	South East	205 (13.4)	15.4
	South West	136 (8.9)	9.4
	West and East Midlands	218 (14.3)	18.1
	Yorkshire and the Humber and North East	197 (12.9)	14
	North West	184 (12)	12.6
	Scotland	129 (8.4)	N/A^a^
	Wales	62 (4.1)	5.5
**General Data Protection Regulation awareness**			
	Not aware	179 (11.7)	—^b^
	Yes, I have heard but do not know much about it	428 (27.9)	—
	Yes, I have heard and know a little about it	658 (42.9)	—
	Yes, I have heard and I know a lot about it	269 (17.5)	—
**Previous health research participation**			
	Yes	402 (26.2)	—
	No	1132 (73.8)	—

^a^N/A: not applicable.

^b^Data are not available for the distribution of the General Data Protection Regulation Awareness and previous health research participation in England.

### GDPR and Personal Data Awareness

At the time of the survey, 11.7% (179/1534) of the participants indicated that they were not aware of GDPR, 27.9% (428/1534) had heard of GDPR but did not know much about it, 42.9% (658/1534) had heard and knew a little about GDPR, and 17.5% (269/1534) of respondents had heard and knew a lot about GDPR.

The results of participants’ expectations of what is considered personal data under GDPR showed that >80% (1227/1534) of the participants were able to correctly state common information that was classified as personal information, such as name, age, gender, marital status, and home address and email address. Less than 75% (1150/1534) of participants considered sensitive personal information, such as sexual orientation (1121/1534, 73.1%), religion (1067/1534, 69.6%), criminal records (1100/1534, 71.7%), and health or medical records (1136/1534, 74.1%) as personal data.

Less than two-thirds of the participants expected the various types of information collected on the internet to count as personal data. A quarter of the participants incorrectly stated that web-based purchases (383/1534, 25.0%), location data based on General Packet Radio Service recorded on mobile phones (353/1534, 23%), tracking information on websites (cookies; 383/1534, 25%), social media information (424/1534, 27.6%), and device IDs (353/1534, 23%) were not personal data.

More than 80% (1227/1534) of the participants correctly identified what GDPR law should cover most of the rights that protect personal data. Most of the remaining participants stated that they did not know the right answer ranging from 6.6% (101/1534) to 13.4% (205/1534). Approximately 13.4% (205/1534) did not know that they had the right to erase their data, and 12.3% (189/1534) did not know that they had the right to be informed about the use of their data. Additional details are provided in Multimedia Appendix 1.

### Willingness to Share Commercial Data With Different Institutions

#### Descriptive Results

**Figure 1:**
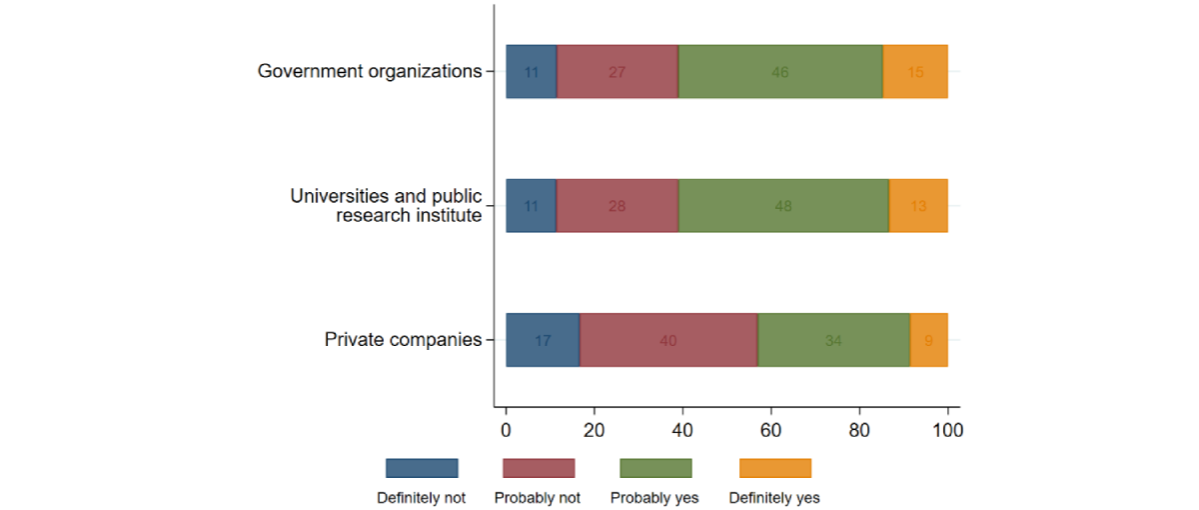
Willingness to share commercial data with different organizations for health research.

Figure 1 shows that nearly two-thirds of the participants indicated that they would be willing to share their commercial data for health research if their data are shared with a government institution (937/1534, 61.1%) or an academic research institution (936/1534, 61.0%). In contrast, less than half were happy to share their commercial data with private organizations for health research (658/1534, 42.9%). Across all participants, only 4.8% (73/1534) of the participants stated “definitely yes” to share with all types of institutions. In comparison, 7.4% (114/1534) of the participants stated “definitely no” to share commercial data for health research with all institutions.

#### Sociodemographic Factors

In Table 2, the analysis shows significant differences in the willingness to share commercial data with government institutions by age, sex, education, and GDPR awareness. Specifically, less than two-thirds of participants were happy to share their data among those aged 40 to 49 years (841/1534, 54.8%) and 50 to 59 years (871/1534, 56.8%), compared with the 18 to 29 (923/1534, 60.2%), 30 to 39 (1040/1534, 67.8%), 60 to 69 (922/1534, 60.1%), and ≥70 years (1020/1534, 66.5%) groups, respectively (*χ*^2^_5_=14.6, *P*=.12). Male participants were more likely to share their commercial data for health research than female participants with the government (994/1534, 64.8% vs 887/1534, 57.8%; *χ*^2^_1_=7.9, *P*=.005). There was a 14.4% difference between willingness to share among those who were unaware of GDPR (822/1534, 53.6%) and those who stated that they knew a lot about GDPR (1043/1534, 68.0%; *χ*^2^_3_=9.7; *P*=.02). No differences were found in marital status, ethnicity, previous research participation, and personal experience of data misuse in the past.

There were differences in the willingness to share commercial data with private organizations based on most factors, except for education and previous research participation. The largest differences were observed for age between those who were aged 30 to 39 years (147/255, 57.6%), and 60 to 69 years (64/213, 30%), and ≥70 years (80/266, 30.1%), as well as by ethnicity among those identified as Black (28/45, 62.2%) and White (555/1326, 41.9%; *χ*^2^_5_=74.1, *P*<.001). Similarly, only one-third of those who reported not being aware of GDPR were willing to share their data (54/179, 30.2%) compared with those who were aware but did not know much (177/428, 41.4%), a little (292/658, 44.4%), and knew a lot about GDPR (138/269, 51.3%; *χ*^2^_3_=20.5, *P*<.001). Ever experienced a negative event of data misuse was also positively associated with willingness to share commercial data for health research with private organizations compared with never experiencing a negative event (287/596, 48.2% vs 374/938, 39.9%; *χ*^2^_1_=10.1, *P*=.001).

There were no significant differences in the proportion of people who indicated “Definitely and Probably yes” to sharing with academic institutions by marital status, age, sex, ethnicity, and previous experience. However, educational level (above degree: 459/697, 65.9% vs below degree: 474/830, 57.1%; *χ*^2^_1_=12.1, *P*<.001), previous participation in health research (yes: 266/402, 66.2% vs no: 670/1132, 59.2%; *χ*^2^_1_=6.0, *P*=.01), and greater GDPR awareness (not aware 84/179, 46.9% vs know a lot about it 179/269, 66.5%; *χ*^2^_3_=20.8, *P*<.001) were positively associated with sharing data with academic institutions.

**Table 2:** Willingness to share commercial data with different organizations by sociodemographic factors (N=1534).

Willingness to share			Government organizations		Private organizations		Academic institutions
**Marital status**							
	Single, n (%)	284 (57.7)		230 (46.7)		306 (62.2)	
	Married or legal partnership, n (%)	512 (62.9)		354 (43.5)		489 (60.1)	
	Widowed, divorced, or separated, n (%)	134 (63.2)		73 (34.4)		134 (63.2)	
	Chi-square (*df*)	3.8 (2)		9.1 (2)		1.0 (2)	
	*P* value	.14		.01		.61	
**Age (years)**							
	18-29, n (%)	171 (60.2)		153 (53.9)		177 (62.3)	
	30-39, n (%)	173 (67.8)		147 (57.6)		168 (65.9)	
	40-49, n (%)	137 (54.8)		118 (47.2)		144 (57.6)	
	50-59, n (%)	151 (56.8)		99 (37.2)		154 (57.9)	
	60-69, n (%)	128 (60.1)		64 (30.0)		129 (60.6)	
	≥70, n (%)	177 (66.5)		80 (30.1)		164 (61.7)	
	Chi-square (*df*)	14.6 (5)		74.1 (5)		5.1 (5)	
	*P* value	.01		<.001		.40	
**Ethnicity**							
	White, n (%)	808 (60.9)		555 (41.9)		821 (61.9)	
	Black, n (%)	33 (73.3)		28 (62.2)		26 (57.8)	
	Asian, n (%)	66 (59.5)		55 (49.5)		64 (57.7)	
	Mixed, n (%)	18 (56.3)		15 (46.9)		15 (46.9)	
	Other ethnicities, n (%)	6		<5		5	
	Chi-square (*df*)	3.2 (4)		9.6 (4)		4.3 (4)	
	*P* value	.51		.04		.35	
**Sex**							
	Male, n (%)	488 (64.8)		359 (47.7)		473 (62.8)	
	Female, n (%)	449 (57.8)		300 (38.6)		462 (59.5)	
	Chi-square (*df*)	7.9 (1)		12.8 (1)		1.8 (1)	
	*P* value	.005		<.001		.17	
**Education**							
	<Degree and no formal education, n (%)	481 (58.0)		367 (44.2)		474 (57.1)	
	≥Degree, n (%)	451 (64.7)		290 (41.6)		459 (65.9)	
	Chi-square (*df*)	7.2 (1)		1.0 (1)		12.1 (1)	
	*P* value	.007		.31		<.001	
**General Data Protection Regulation awareness**							
	Not aware, n (%)	96 (53.6)		54 (30.2)		84 (46.9)	
	Yes, I have heard but do not know much about it, n (%)	258 (60.3)		177 (41.4)		254 (59.3)	
	Yes, I have heard but know little about it, n (%)	400 (60.8)		292 (44.4)		419 (63.7)	
	Yes, I have heard and I know a lot about it, n (%)	183 (68.0)		138 (51.3)		179 (66.5)	
	Chi-square (*df*)	9.7 (3)		20.5 (3)		20.8 (3)	
	*P* value	.02		<.001		<.001	
**Previous research participation**							
	Yes, n (%)	257 (63.9)		171 (42.5)		266 (66.2)	
	No, n (%)	680 (60.1)		490 (43.3)		670 (59.2)	
	Chi-square (*df*)	1.8 (1)		0.1 (1)		6.0 (1)	
	*P* value	.17		.79		.01	
**Personal experience of data misuse**							
	Never, n (%)	559 (59.7)		374 (39.9)		559 (59.6)	
	Ever, n (%)	378 (63.4)		287 (48.2)		377 (63.3)	
	Chi-square (*df*)	2.2 (1)		10.1 (1)		2.0 (1)	
	*P* value	.13		.001		.15	

#### Psychological Predictors

Adjusted ordered regression analyses for willingness to share data with each institution in Table 3 show that greater trust is positively associated with sharing commercial data with government (aOR 2.499, 95% CI 2.228-2.802; *P*<.001), private (aOR 2.513, 95% CI 2.221-2.842; *P*<.001), and academic institutions (aOR 2.283, 95% CI 2.011-2.59; *P*<.001). Greater worry about data misuse was negatively associated with willingness to share with government (aOR 0.94, 95% CI 0.918-0.961; *P*<.001), private (aOR 0.951, 95% CI 0.930-0.973; *P*<.001), and academic institutions (aOR 0.947, 95% CI 0.926-0.969; *P*<.001).

Participants’ perceived importance of privacy was negatively associated with willingness to share with the government (aOR 0.909, 95% CI 0.833-0.992; *P*=.03), private institutions (aOR 0.833, 95% CI 0.763-0.909; *P*<.001), and academic institutions (aOR 0.869, 95% CI 0.797-0.948; *P*=.002). Participants’ perceived risk of data sharing was not associated with their willingness to share their data with any organization. The perceived benefits of sharing data were positively associated with government institutions (aOR 1.111, 95% CI 1.083-1.14; *P*<.001), private institutions (aOR 1.081, 95% CI 1.054-1.109; *P*<.001), and academic institutions (aOR 1.116, 95% CI 1.087-1.146; *P*<.001).

**Table 3:** Psychological predictors of willingness to share commercial data for health research with different organizations^a^.

	Government institutes, aOR^b^ (95% CI)	Private institutes, aOR (95% CI)	Academic institutes, aOR (95% CI)
Trust in organizations	2.499 (2.228-2.802^c^)	2.513 (2.221-2.842^c^)	2.283 (2.011-2.590^c^)
Worry about data misuse	0.940 (0.918-0.961^c^)	0.951 (0.930-0.973^c^)	0.947 (0.926-0.969^c^)
Perceived risk of data sharing	1.041 (0.997-1.086)	1.042 (0.997-1.089)	1.016 (0.974-1.060)
Perceived importance of privacy	0.909 (0.833-0.992^d^)	0.833 (0.763-0.909^c^)	0.869 (0.797-0.948^c^)
Perceived benefits of sharing data and participation	1.111 (1.083-1.140^c^)	1.081 (1.054-1.109^c^)	1.116 (1.087-1.146^c^)

^a^Adjusted for age, sex, location, ethnicity, education, General Data Protection Regulation awareness, and past health research participation. The full model with *P* values is reported in Multimedia Appendix 1.

^b^aOR: adjusted odds ratio.

^c^*P*<.001.

^d^*P*<.05.

### Willingness to Share Different Types of Commercial Data

#### Descriptive Results

**Figure 2:**
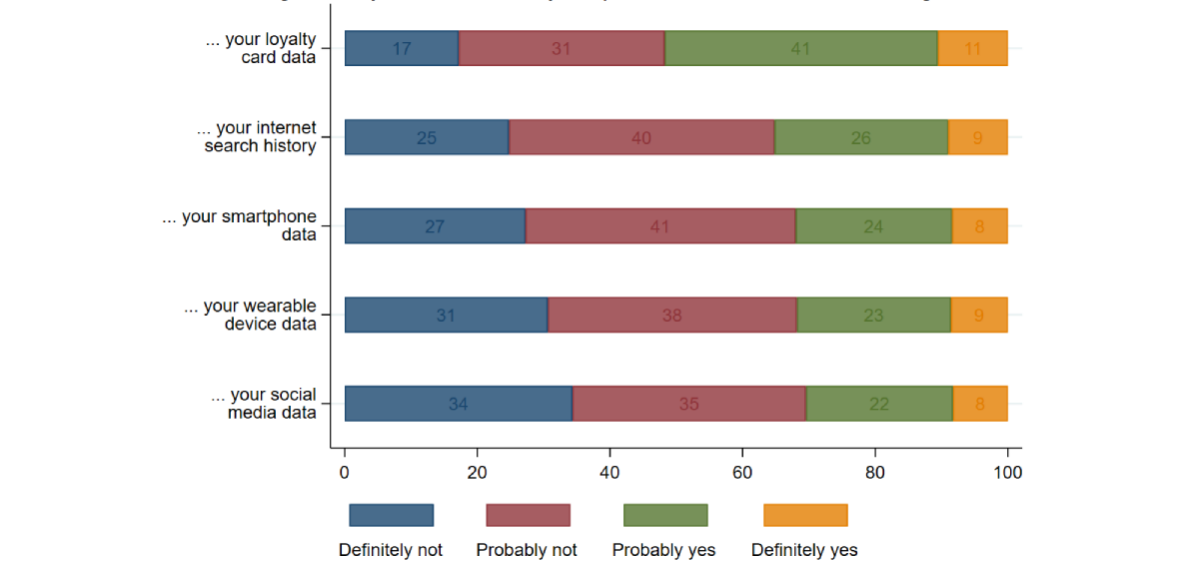
Willingness to share different types of commercial data with academic institutions for health research.

Figure 2 shows that the participants’ willingness to share commercial data varied across all data types. The willingness to share loyalty card data had the highest proportion of participants at 51.8% (795/1534) stating that “Definitely or Probably yes.” In comparison, the proportion was much lower at 35% (540/1534) for internet search history, 32% (491/1534) for smartphone data, 32% (488/1534) for sharing wearable device data, and 30% (467/1534) for social media data. Across all participants, only about 3.2% (49/1534) of the participants stated “definitely yes” to share all types of commercial data sets. In comparison, 13.3% (204/1534) of the participants stated “definitely no” to share all types of commercial data sets.

#### Sociodemographic Factors

Table 4 shows the proportion of people who stated “Definitely and Probably yes” for willingness to share different types of commercial data for health research with academic institutions based on the sociodemographic characteristics of the participants. There were significant differences across all types of health research data according to marital status, age, and past experience of data misuse. In contrast, no associations were found between the participants’ educational level and previous participation in the research. Greater GDPR awareness was positively associated with willingness to share all types of data, except for internet searches.

Among these characteristics, notable differences were observed for marital status, where a larger proportion of people who were single reported willingness to share commercial data sets compared with those who were married or in a legal partnership, or widowed, divorced, or separated. Furthermore, an increase in the age of participants was negatively associated with their willingness to share. Less than a fifth of the participants in the 60 to 69 years and above age groups were willing to share smartphone, social media, and wearable device data. Across all types of commercial data, those aged 18 to 29 years had the highest proportion of individuals willing to share at 65.1% (185/284) for loyalty card data, 48.6% (138/284) for smartphone data and wearable devices, 47.2% (134/284) for social media, and 46.8% (133/284) for internet data.

Female participants were less likely to share smartphone data (male: 282/753, 37.5% vs female: 207/777, 26.6%; *χ*^2^_1_=20.5 *P*<.001), wearable devices (male: 280/753, 37.2% vs female: 206/777, 26.5%; *χ*^2^_1_=20.0 *P*<.001), and social media data (male: 271/753, 36% vs female: 196/777, 25.2%; *χ*^2^_1_=20.8 *P*<.001).

In comparison, the differences in proportions were smaller for internet searches (male: 286/753, 38% vs female: 253/777, 32.6%; *χ*^2^_1_=4.9, *P*=.03) and loyalty card data (male: 399/753, 53% vs female: 393/777, 50.6%; *χ*^2^_1_=0.8, *P*=.35). The proportion of people willing to share loyalty card data did not differ by ethnicity or sex. In contrast, the proportion was lower among those from White ethnic backgrounds for internet searches, social media, wearable devices, and smartphone data compared with those who were identified from Black and Asian ethnic backgrounds.

Those who had ever experienced a data misuse event were more likely to share loyalty card data (ever: 344/596, 57.7% vs never: 450/938, 48%; *χ*^2^_1_=13.8, *P*<.001), internet search data (ever: 263/596, 44.1% vs never: 277/938, 29.5%; *χ*^2^_1_=34.0, *P*<.001), smartphone (ever: 245/596, 41.1% vs never: 246/938, 26.2%; *χ*^2^_1_=37.0, *P*<.001), social media (ever: 240/596, 40.3% vs never: 227/938, 24.2%; *χ*^2^_1_=44.4, *P*<.001), and wearable devices (ever: 255/596, 42.8% vs never: 233/938, 24.8%; *χ*^2^_1_=54.1, *P*<.001) than those with no previous data misuse experience.

**Table 4:** Willingness to share different types of commercial data by sociodemographic factors.

Willingness to share				Internet searches		Loyalty card		Smartphone		Social media		Wearable devices
**Marital status**												
	Single, n (%)		205 (41.7)		297 (60.4)		190 (38.6)		180 (36.6)		194 (39.4)	
	Married or legal partnership, n (%)		273 (33.5)		406 (49.9)		263 (32.3)		235 (28.9)		250 (30.7)	
	Widowed, divorced, or separated, n (%)		57 (26.9)		87 (41.0)		34 (16.0)		48 (22.6)		41 (19.3)	
	Chi-square (*df*)		16.4 (2)		25.4 (2)		34.7 (2)		15.7 (2)		28.7 (2)	
	*P* value		<.001		<.001		<.001		<.001		<.001	
**Age (years)**												
	18-29, n (%)		133 (46.8)		185 (65.1)		138 (48.6)		134 (47.2)		138 (48.6)	
	30-39, n (%)		132 (51.8)		156 (61.2)		122 (47.8)		129 (50.6)		141 (55.3)	
	40-49, n (%)		98 (39.2)		134 (53.6)		94 (37.6)		81 (32.4)		87 (34.8)	
	50-59, n (%)		70 (26.3)		129 (48.5)		66 (24.8)		60 (22.6)		57 (21.4)	
	60-69, n (%)		45 (21.1)		91 (42.7)		34 (16.0)		29 (13.6)		29 (13.6)	
	≥70 , n (%)		62 (23.3)		99 (37.2)		37 (13.9)		34 (12.8)		36 (13.5)	
		Chi-square (*df*)	93.4 (5)		60.3 (5)		140.4 (5)		162.3 (5)		189.4 (5)	
		*P* value	<.001		<.001		<.001		<.001		<.001	
**Ethnicity**												
	White, n (%)		447 (33.7)		674 (50.8)		400 (30.2)		377 (28.4)		397 (29.9)	
	Black, n (%)		25 (55.6)		26 (57.8)		22 (48.9)		25 (55.6)		24 (53.3)	
	Asian, n (%)		53 (47.7)		66 (59.5)		50 (45.0)		47 (42.3)		49 (44.1)	
	Mixed, n (%)		9 (28.1)		20 (62.5)		13 (40.6)		13 (40.6)		15 (46.9)	
	Other ethnicities, n (%)		5 (50.0)		6 (60.0)		<5		<5		<5	
		Chi-square (*df*)	18.7 (4)		5.4 (4)		17.9 (4)		24.8 (4)		23.4 (4)	
		*P* value	.001		.24		.001		<.001		<.001	
**Sex**												
	Male, n (%)		286 (38.0)		399 (53.0)		282 (37.5)		271 (36.0)		280 (37.2)	
	Female, n (%)		253 (32.6)		393 (50.6)		207 (26.6)		196 (25.2)		206 (26.5)	
		Chi-square (*df*)	4.9 (1)		0.8 (1)		20.5 (1)		20.8 (1)		20.0 (1)	
		*P* value	.02		.34		<.001		<.001		<.001	
**Education**												
	<Degree and no formal education, n (%)		290 (34.9)		435 (52.4)		252 (30.4)		244 (29.4)		248 (29.9)	
	≥Degree, n (%)		247 (35.4)		355 (50.9)		235 (33.7)		219 (31.4)		238 (34.1)	
		Chi-square (*df*)	0.4 (1)		0.3 (1)		1.9 (1)		0.7 (1)		3.1 (1)	
		*P* value	.83		.56		.16		.39		.07	
**General Data Protection Regulation awareness**												
	Not aware, n (%)		52 (29.1)		74 (41.3)		49 (27.4)		39 (21.8)		48 (26.8)	
	Yes, I have heard but do not know much about it, n (%)		159 (37.1)		216 (50.5)		129 (30.1)		136 (31.8)		129 (30.1)	
	Yes, I have heard but know little about it, n (%)		221 (33.6)		351 (53.3)		196 (29.8)		192 (29.2)		197 (29.9)	
	Yes, I have heard and I know a lot about it, n (%)		108 (40.1)		153 (56.9)		117 (43.5)		100 (37.2)		114 (42.4)	
		Chi-square (*df*)	7.3 (3)		11.5 (3)		20.2 (3)		12.9 (3)		17.5 (3)	
		*P* value	.06		.009		<.001		.005		.001	
**Previous research participation**												
	Yes, n (%)		131 (32.6)		195 (48.5)		122 (30.3)		115 (28.6)		114 (28.4)	
	No, n (%)		409 (36.1)		599 (52.9)		369 (32.6)		352 (31.1)		374 (33.0)	
		Chi-square (*df*)	1.6 (1)		2.3 (1)		0.6 (1)		0.8 (1)		2.9 (1)	
		*P* value	.20		.12		.40		.35		.08	
**Personal experience of data misuse**												
	Never, n (%)		277 (29.5)		450 (48.0)		246 (26.2)		227 (24.2)		233 (24.8)	
	Ever, n (%)		263 (44.1)		344 (57.7)		245 (41.1)		240 (40.3)		255 (42.8)	
		Chi-square (*df*)	34.0 (1)		13.8 (1)		37.0 (1)		44.4 (1)		54.1 (1)	
		*P* value	<.001		<.001		<.001		<.001		<.001	

#### Psychological Predictors

The results from the ordered logistic regression analyses adjusted for the sociodemographic characteristics of the participants in Table 5 show that each point increase in trust in data practices in academia, perceived benefits in participation, and perceived risks of data sharing are positively associated with willingness to share all types of commercial data. In contrast, each point increase in the perceived importance of privacy and worry about data misuse was negatively associated with the willingness to share all types of commercial data.

**Table 5:** Psychological predictors of willingness to share different types of commercial data for health research with academic institutions^a^.

	Internet search data, aOR^b^ (95% CI)	Loyalty card data, aOR (95% CI)	Smartphone data, aOR (95% CI)	Social media data, aOR (95% CI)	Wearable devices data, aOR (95% CI)
Trust in data practices in academic institutions	1.097 (1.078-1.117^c^)	1.103 (1.083-1.123^c^)	1.097 (1.077-1.117^c^)	1.087 (1.067-1.107^c^)	1.075 (1.056-1.095^c^)
Perceived importance of privacy	0.682 (0.625-0.744^c^)	0.707 (0.648-0.772^c^)	0.685 (0.628-0.748^c^)	0.716 (0.656-0.781^c^)	0.732 (0.670-0.798^c^)
Worry about data misuse	0.960 (0.938-0.982^c^)	0.975 (0.953-0.997^d^)	0.960 (0.938-0.982^c^)	0.963 (0.941-0.985^c^)	0.940 (0.919-0.962^c^)
Perceived benefit in data sharing and research participation	1.057 (1.029-1.086^c^)	1.102 (1.072-1.132^c^)	1.070 (1.040-1.100^c^)	1.049 (1.020-1.078^c^)	1.086 (1.056-1.116^c^)
Perceived risks of data sharing	1.222 (1.169-1.276^c^)	1.114 (1.067-1.163^c^)	1.224 (1.171-1.279^c^)	1.208 (1.156-1.263^c^)	1.232 (1.179-1.287^c^)

^a^Adjusted for age, sex, location, ethnicity, education, General Data Protection Regulation awareness, and past health research participation. The full model with *P* values is reported in Multimedia Appendix 1.

^b^aOR: adjusted odds ratio.

^c^*P*<.001.

^d^*P*<.05.

### Willingness to Take Part in Research Based on Invitation Sources

**Figure 3:**
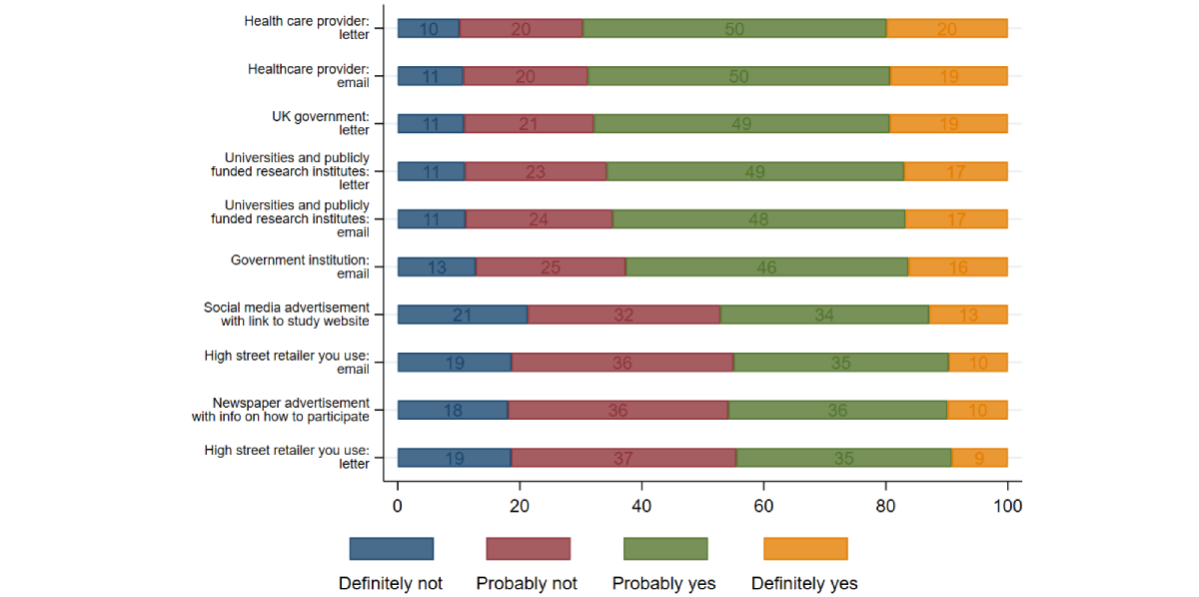
Preferences for the source of research participation invitation.

Figure 3 shows that the most preferred ways of being invited to health research using commercial data were receiving a letter (1069/1534, 69.6%) or an email invitation (1056/1534, 68.6%) from the health care provider, followed by a letter invitation from the government (1041/1534, 67.9%) and universities or publicly funded research institutes (1009/1534, 65.8%). Digital invitations had a much lower preference compared with letter-based invitations, except for letter invitations from high-street retailers to participate in the research (683/1534, 44.5%). Research advertisements on social media or newspapers were preferable for less than half of the participants, with 47.1% (723/1534) and 45.8% (703/1534), respectively. The ordered logistic regression analysis in Multimedia Appendix 1 shows that across all invitation types, greater GDPR awareness was the only predictor of invitation source, and there were some nuanced differences in sociodemographic characteristics.

## Discussion

### Principal Findings

With an interest to develop a better understanding of psychological factors and sociodemographic characteristics of individuals who would be willing to participate in health research using individual and commercially collected data sets, this study investigates the willingness to share commercial data for health research with different institutions and different types of commercial data in an age-stratified population-based sample in the United Kingdom. Our results showed that two-thirds of the participants were willing to share their commercial data on health research with academic institutions. In contrast, when participants were specifically asked about sharing different types of commercial data for health research with a focus on academic institutions, only about half of them were willing to share their shopping data, and less than one-third were happy to share internet search history, wearable devices, social media, and smartphone apps. Only a small minority of the participants across all outcomes were willing to share their data, highlighting the potential barrier in participant recruitment that needs to be addressed in health research using complex data sets.

### Comparison With Prior Work

A key outcome of this study is the validation of the previous evidence that, irrespective of individuals’ GDPR awareness and their sociodemographic characteristics, greater trust is consistently associated with greater willingness to share commercial data for health research [12]. This study also adds to the evidence that greater perceived importance of data privacy and greater worry about data misuse are negatively associated with the willingness to share commercial data for health research. Interestingly, the perceived risk of sharing data was not associated with the willingness to share data with institutions, but it played a role in all types of data that they were willing to share. This is in line with the Nissenbaum [29] contextual integrity framework for privacy, suggesting that individuals’ information-sharing principles are context dependent and socially constructed. A previous focus group study also found similar results in that participants were more concerned about the use of subjective data sets such as social media posts compared with objective shopping data collected on loyalty cards as more important than the organizations in which they share their data with [15]. Furthermore, we found a positive univariate association between the participants who had ever experienced a negative data-related event, for example, they might have had a data breach and personal information stolen on the internet and used for other people’s gain, were more likely to share their data. Although we do not have sufficient information to assess why this may be the case, it could be interpreted that those who have experienced a negative event are more likely to be risk-aware than risk-averse. An interesting explanation for this result could be studied further based on “the privacy paradox,” which suggests that intentions to share data may not be directly associated with actual behavior, and other mediators should be better understood [30]. Future experimental studies with a factorial design can further explore how these experiences impact people’s perceptions and use of technologies.

We identified various sociodemographic factors associated with all outcome variables with the participants’ age particularly being an important factor to be considered for participant recruitment. Our results showed that the participants’ willingness reduced with an increase in age, except for sharing data with government institutions where we observed an inverse association. This is an important outcome to consider when implementing pilot health interventions for the general population [17]. The participants who identified themselves as Black consistently had a higher proportion of willingness not just across all institutions but also for all data types. Health researchers should identify resources to improve the visibility of health research opportunities to improve participation and diversity in research. Recruitment through social media advertisements has been shown to be effective in targeting minority populations [31]. However, there is a lack of ethical and methodological guidance for recruitment via paid social media advertisements to be carried out effectively [32].

### Limitations

A key limitation of this survey was that participants were not provided with examples of how commercial data could be used for specific health studies, such as facilitating earlier cancer diagnosis, identifying mental health conditions, and not including nonprofit organizations. A previous study showed that people were more willing to donate their data to Cancer Research UK compared with nonspecific health research organizations, which was found to be associated with individuals’ level of altruism and prosocial tendencies [33]. Owing to the exploratory nature of this study, a priori hypotheses were not included in the statistical analysis plans, and the following warnings are warranted. Although we adjusted for past health research participation, participants from the recruitment panel were subject to desirability bias. The social desirability bias in perceived privacy, intention to share data, and actual behaviors has previously been demonstrated in the privacy paradox [30]. Future studies arising from this study could investigate mediators between intention and behavior gaps. Similarly, the use of nonprobability sampling of the participants could also lead to greater bias in this study. Although accumulating evidence suggests that the willingness to share commercial data is a complex behavior and cannot be reduced to one-off intention measures, we believe that the outcomes of this study can be used as a guide for identifying populations with the least likelihood of sharing data when recruitment methods for studies requesting data from its participants are operationalized. However, it should be acknowledged that the measures included in this study, as well as sociodemographic characteristics, explain less than 20% of the variance in the willingness to share commercial data (Multimedia Appendix 1). This highlights the complexity of the evidence surrounding data sharing and slow progress in health-related research in relation to building a better understanding of the mechanisms that hinder and facilitate data-sharing principles. Thus, future studies could benefit from the use of a theoretical model, such as the capability, opportunity, motivation, and behavior model [34], to understand how physical and psychological capabilities, such as participants’ engagement with existing technologies, could potentially moderate their willingness to share data. Similarly, social opportunities could be an important factor for the willingness to participate in research advertised through social media. Notwithstanding, this survey included a UK age-representative cohort based on the 2011 Census [26], had a larger proportion of individuals from non-White ethnic backgrounds in comparison with other panel-based survey studies with a UK population representative sample [35,36], and also adjusted for the participants’ geographic region in the United Kingdom to improve the external validity and generalizability of its outcomes for the wider UK population.

### Conclusions

This survey study demonstrated the public acceptability of sharing commercial data for health research in the United Kingdom with an extensive exploration of people’s knowledge and understanding of what constitutes personal data and GDPR, their willingness to share with different organizations, their willingness to share various types of commercial data, and their willingness to consent and share data if invited through different methods. The outcomes of this study are of interest to be considered in the guidelines and recommendations for public acceptability of data sharing beyond electronic health records and will be useful for developing data stewardship frameworks and initiatives to improve the use of data in the United Kingdom. Where possible, these outcomes can also be used to develop recruitment strategies for research using stratified sampling techniques where it is expected to have low response rates. Future studies using experimental methods are warranted to identify the effectiveness of behavioral science techniques and communication methods to improve the public acceptability of sharing commercial data for health research.

## Appendix

Multimedia Appendix 1 (1) The results of the factor analysis; (2) a detailed description of the survey measures including the original questionnaire with item heritage; (3) descriptive data tables for the General Data Protection Regulation awareness in the United Kingdom; (4) ordered logistic regression results on willingness to share commercial data based on invitation sources; and (5) full ordered regression model tables for the primary outcomes.

## Abbreviations

aOR: adjusted odds ratio
GDPR: General Data Protection Regulation
PCA: principal component analysis

## Data Availability

The anonymized data collected as part of this survey will be made available to researchers upon publication in the Open Science Framework.
