# Supplementary material for: Understanding Public Attitudes and Willingness to Share Commercial Data for Health Research: Survey Study in the United Kingdom
Source: JMIR Public Health Surveill. 2023 Mar 23;9:e40814. doi: 10.2196/40814 (PMC10131900; doi:10.2196/40814)
Supplement: Multimedia Appendix 1 [file publichealth_v9i1e40814_app1.docx]

Appendix

# Factor Analysis Results

## Factor Analysis Results

The 32 Items measured in the study were subjected to Principal Components analysis (PCA) using SPSS version 27. Prior to performing PCA the Suitability for performing PCA was assessed. The inspection of correlation matrix revealed the presence of many coefficients above 0.3. The Kaiser-Meyer-Olkin value was 0.93 above the recommended value of 0.6. and Bartlett’s Test of Sphericity reached statistical significance supporting the factorability of the correlation matrix. The principal components analysis revealed the presence of 6 components with eigenvalues exceeding 1, explaining, 28.6%, 19.7%, 7.5%, 6,9%, 5.3% and 3.2% variance respectively (Supplementary Table 1). Factor correlations as separate scales suggests the scales have week to moderate correlation indicating they are measuring separate scales (See supplementary Table 2). 8 Items were recoded and two Items were deleted as they measured trust in two different organisations.

Supplementary Table 1 Pattern Matrix results using Principal Components Analysis with action plan

| Items included | |  | | | | |
| --- | --- | --- | --- | --- | --- | --- |
|  |  | 1 | 2 | 3 | 4 | 5 |
| TRUST in data practices in academia | The way scientists in universities and publicly funded research institutes make the decisions on how to handle the public’s personal data is fair. | .887 |  |  |  |  |
|  | Scientists in universities and publicly funded research institutes have the skills and expertise to handle the public’s personal data in a way that protects their privacy. | .883 |  |  |  |  |
|  | The way scientists in universities and publicly funded research institutes make the decisions to handle the public’s personal data is transparent. | .874 |  |  |  |  |
|  | Scientists in universities and publicly funded research institutes are fair in their user policies regarding the use of the public’s personal data. | .874 |  |  |  |  |
|  | Scientists in universities and publicly funded research institutes have access to the information and skills to handle the public’s personal data securely. | .872 |  |  |  |  |
|  | Scientists in universities and publicly funded research institutes keep the public’s best interest in mind when handling their data. | .852 |  |  |  |  |
|  | I trust research carried out by academic institutions Recode  (Single Item for Trust but also included in the total score) | -.819 |  |  |  |  |
|  | Scientists in universities and publicly funded research institutes have the same opinions as me about handling the public's data. | .794 |  |  |  |  |
|  | I would feel safe giving my data to scientists in universities and publicly funded research institutes. | .752 |  |  |  |  |
|  | I trust research carried out by the government Recode (Exclude) – Single Item | -.561 |  |  |  |  |
|  | I trust research carried out by the commercial organisations Recode (Exclude) -Single Item | -.540 |  |  | .370 |  |
| Worry about data misuse | I would worry organizations might use my information for purposes not stated in the consent or privacy policy. |  | .921 |  |  |  |
|  | I would worry my personal data will be shared to others without my consent. |  | .906 |  |  |  |
|  | I would worry about the intention of the data collection. |  | .902 |  |  |  |
|  | I would worry about the abuse of my data. |  | .877 |  |  |  |
|  | I would worry someone would use my personal data to make money. |  | .875 |  |  |  |
|  | I would worry about the privacy of my data. |  | .831 |  |  |  |
| Perceived benefits of participating health research | My participation could help my family |  |  | .900 |  |  |
|  | My participation could lead to better medical treatments. |  |  | .870 |  |  |
|  | My participation could improve the patients' health outcomes. |  |  | .869 |  |  |
|  | My participation could help me personally |  |  | .860 |  |  |
|  | My participation could help future generations. |  |  | .847 |  |  |
| Perceived risks of data donation | There would be too much uncertainty associated with giving my data to scientists in universities and publicly funded research institutes. (Recode) |  |  |  | -.883 |  |
|  | In general, it would be risky to give my data to scientists in universities and publicly funded research institutes. (Recode) |  |  |  | -.882 |  |
|  | Providing scientists in universities and publicly funded research institutes with my data would involve many unexpected problems. (Recode) |  |  |  | -.851 |  |
| Perceived Importance of privacy | Data privacy is important to me (Recode) |  |  |  |  | -.811 |
|  | I feel uncomfortable when other people have access to my personal data (Recode) |  |  |  |  | -.797 |

| Supplementary Table 2. The Correlations between the factors once converted into scales | | | | | | |
| --- | --- | --- | --- | --- | --- | --- |
|  | | Importance of privacy (.65) | Perceived risk of data donation (Alpha .89) | Worry (6 items) Alpha .95 | Total trust (9 items) Alpha .95 | Total perceived benefit of participation (.93) |
| Importance of privacy (.65) | Pearson Correlation | 1 | .231^**^ | .493^**^ | .024 | .110^**^ |
|  | Sig. (2-tailed) |  | .000 | .000 | .357 | .000 |
|  | N | 1534 | 1534 | 1534 | 1534 | 1534 |
| Perceived Risk of data donation (Alpha .89) | Pearson Correlation | .231^**^ | 1 | .428^**^ | .008 | .054^*^ |
|  | Sig. (2-tailed) | .000 |  | .000 | .755 | .036 |
|  | N | 1534 | 1534 | 1534 | 1534 | 1534 |
| Worry about data misuse  (6 items) Alpha .95 | Pearson Correlation | .493^**^ | .428^**^ | 1 | -.176^**^ | .069^**^ |
|  | Sig. (2-tailed) | .000 | .000 |  | .000 | .007 |
|  | N | 1534 | 1534 | 1534 | 1534 | 1534 |
| Total trust in data practices in academia (9 items) Alpha .95 | Pearson Correlation | .024 | .008 | -.176^**^ | 1 | .493^**^ |
|  | Sig. (2-tailed) | .357 | .755 | .000 |  | .000 |
|  | N | 1534 | 1534 | 1534 | 1534 | 1534 |
| Total perceived benefit of participation (.93) | Pearson Correlation | .110^**^ | .054^*^ | .069^**^ | .493^**^ | 1 |
|  | Sig. (2-tailed) | .000 | .036 | .007 | .000 |  |
|  | N | 1534 | 1534 | 1534 | 1534 | 1534 |
| **. Correlation is significant at the 0.01 level (2-tailed). | | | | | | |
| *. Correlation is significant at the 0.05 level (2-tailed). | | | | | | |

Supplementary section 2: Survey Measures included in the final analysis

### ***Socio-Demographic Factors***

**Participants were asked to provide personal information about themselves including their age range (18-29/30-39/40-49/50-59/60-69/70+), gender (male/female/other), educational level (Higher Education or professional and vocational equivalents/ A-levels, vocational level 1-3, GCSE, O Level grade A-C, or level unknown including foreign qualifications/no qualification), marital status (single/married & in legal partnership/widowed, divorced, separated/preferred not to say), ethnicity (Black/White/Asian/Mixed/Other/prefer not to say), living area (London/East of England/South East/South West/West Midland/ Yorkshire and Humber/North West/ North East/ Scotland/Wales/ Prefer not to say).**

### ***GDPR awareness***

**Participants' awareness of the GDPR was measured using a single awareness item that aimed to understand their level of engagement since it came into effect in 2018 using four statements (“No, not aware”/ “Yes, I have heard about it, but do not know much about it”/ “Yes, I have heard and I know a little about it”/ “Yes, I have heard and I know a lot about it”).**

### ***Expectations of personal data under GDPR***

**Participants were asked to state from a list of data categories for the things they would expect to count as personal data under GDPR. Three options were given for each category (“Yes”, “No”, and “I don’t know”). The questions were adopted from the GDPR OnLineBus Survey (2018) [23].**

### ***Expectations of what GDPR law covers***

**We asked participants to choose from a list of rights that they would expect the GDPR law to cover based on the seven GDPR rights given to the public including lawfulness, fairness and transparency, purpose limitation, data minimization, accuracy, storage limitation, integrity and confidentiality, and accountability (Information Commissioner’s Office). Participants were asked to select from three choices: “Yes”, “No”, and “I don’t know”. The questions were adopted from the GDPR OnLineBus Survey (2018) [23].**

### ***Willingness to share commercial data with different organizations***

**Participants were asked to state their willingness to share commercial data with different organizations based in the UK including governments, private companies, universities and publicly-funded research institutes ranging from “Definitely no”/”Probably no”/”Probably yes”/”Definitely yes”.**

### ***Willingness to share different types of commercial data***

**The survey also asked participants for their willingness for sharing different types of commercial data including internet searches, buying habits collected using loyalty cards from high street retailers, smartphone applications that collect lifestyle and behaviour information, and social media posts (e.g., Facebook, Twitter etc.), and wearable devices (e.g. Fitbit, Garmin etc).**

### ***Trust in Organisations***

**Trust in organisations was measured using a single item “I trust research carried out by…” for each organisation (Government/Commercial Organisations/ academic institutions) using a 5-point Likert scale ranging from strongly agree to strongly disagree.**

### ***Trust in data practices in Academic Institutions (9 Items [Range 5-45], Mean (SD)= 31.96 (7.14), Cronbach Alpha= .95)***

**Trust in data practices were measured with 9 items with a 5-point likert scale ranging from Strongly agree to Strongly disagree. The questions were developed based on Bhattacherjee’s study (2002) about individual trust [24] and Jarvenpaa et al.’s study (1999) about consumer trust in an internet store [25] and were adapted to measure trust in information governance practices in academic institutions.**

### ***Perceived Importance of Data Privacy* ([Range 2-10], Mean (SD)=8.46 (1.38), *Cronbach alpha= .65),***

**The perceived importance of data privacy was measured with two items using a 5-point Likert scale ranging from Strongly Agree to Strongly Disagree. A total score was calculated for the total perceived importance of data privacy.**

### Worry about data misuse (6 Items, [Range 5-30] Mean (SD)= 21.28 (5.69), Cronbach Alpha= .95)

Participants were asked to rate to what extent they had perceived concerns about taking part in health research based on items that included statements on potential data misuse (Always/ Often/ Sometimes/ Rarely/ Never). The questions were adopted from Sanderson et al. ’s (2017) research that studied people’s perceived concerns for sharing genetic information [26]*.*

### Perceived risk of data donation for health research (3 Items, [Range 3-15], Mean (SD)= 9.40 (2.73), Cronbach Alpha= 0.88)

Three items measured individuals’ perception of risks of giving data for health research using a 5-point Likert scale ranging from Strongly agree to strongly disagree. Items included statements such as “In general, it would be too risky to give my data to the scientists in universities and publicly funded research institutes”

### Perceived Benefits of data sharing ( 5 Items, Range 5-25, Mean(SD)= 17.76 (4.31), Cronbach Alpha= .93)

Five items about benefits were given in this section to ask participants to what extent they perceived their participation will be beneficial for themselves, family members or the public. The questions were adapted from Seltzer et al (2019)’s study about patients’ willingness to share commercial data with health research [27]. Five options were provided including “Always”, “Often”, “Sometimes”, “Rarely”, and “Never”*.*

### Previous experience of data misuse

Participants were also asked to indicate if any of the listed commercial data-related crimes had happened to them online. Three choices were given for each situation: “Yes”, “No”, and “Unsure”.

### Previous Health Research Participation

We asked the participants to indicate if they have ever taken part in health-related research before with a single categorical item (“Yes”/”No”).

### Willingness to take part in research based on invitation sources

Participants were asked to consider ten different pathways where they may receive an invitation to take part in health research and consent to share commercial data. These pathways were selected based on the most common methods in health research and were split into four different modes of invitations such as postal, email, social media and newspaper invitations and to be considered by four different organisations including government, health care providers, private institutions, and academic institutions. Participants' responses to their willingness to take part in research based on the type of invitation were measured with “Definitely no”/”Probably no”/ “Probably yes”/”Definitely yes”.

Supplementary Material Section 3: Original Questionnaire with Item Heritage

| Topic | Question | Sub-question | Scale | Heritage |
| --- | --- | --- | --- | --- |
| Awareness of GDPR | Q1: “General Data Protection Regulation” | Q1-1:Have you heard of General Data Protection Regulation (GDPR)? | --No, not aware  --Yes, I have heard but do not know much about it  --Yes, I have heard and I know a little about it.  --Yes, I have heard and I know a lot about it. |  |
|  |  | Q1-2: Since GDPR came into effect in 2018, to what extent have you become aware of the way your personal data is handled by different organisations? | --None at all  --A little  --Somewhat  --A lot  --Extremely |  |
|  |  | Q1-3: Since GDPR came into effect, have you stopped using a service or buying from an organisation? | --Yes  --No |  |
| Personal Data Under GDPR | Q2: Which of these things do you expect to count as your personal data under GDPR? | Name | --Yes  --No  --I don’t know | GDPR OnLineBus Survey (S7304 - 260413108) |
|  |  | Age |  |  |
|  |  | Gender |  |  |
|  |  | Marital Status |  |  |
|  |  | Sexual Orientation |  |  |
|  |  | Religion |  |  |
|  |  | Home Address |  |  |
|  |  | Criminal Record |  |  |
|  |  | Email Address |  |  |
|  |  | Health/medical Records |  |  |
|  |  | Tracking data about which websites I have visited |  |  |
|  |  | Device IDs which enable companies or organization to send information personally to my computer or smartphone. |  |  |
|  |  | Information on products and services I have bought online. |  |  |
|  |  | Information on my interests based on things I have linked on social media. |  |  |
|  |  | Information on my location based on GPRS data from my smartphone. |  |  |
| GDPR Law | Q3: Which of these things would you expect the GDPR law to cover? | Companies / organisations need to clearly ask for my permission to use my personal data. | --Yes  --No  --I don’t know | GDPR OnLineBus Survey (S7304 - 260413108) |
|  |  | I should be able to ask companies / organisations to stop marketing to me directly. |  |  |
|  |  | Companies / organisations need to guarantee that any data they hold on me is not passed onto any other companies / organisations without my consent. |  |  |
|  |  | Companies / organisations must notify me immediately of any breach of my personal data (that is, if my data is accessed from anyone outside the organisation). |  |  |
|  |  | I can ask companies / organisations to erase any data they hold about me. |  |  |
|  |  | I can ask companies / organisations to tell me what data they hold about me. |  |  |
|  |  | Companies / organisations need to use plain and simple language to explain how they want to use my data. |  |  |
|  |  | Companies / organisations must ask me to opt into them using my personal data rather than expecting me to opt out. |  |  |
|  |  | Companies / organisations should pay heavy fines to the government in the event of any data breach. |  |  |
|  |  | Companies / organisations should compensate me in the event of any data breach. |  |  |
|  |  | Companies / organisations should pay me or offer me goods and services in return for using my personal data. |  |  |
| Willingness to share your commercial data for health research | Q4. Organisations obtain personal information about individuals from a variety of different sources. Internet searches, buying habits, lifestyle and behaviour data gathered from mobile phones, social networks, video surveillance systems are examples of the types of data organisations might collect. These can be classified as special category data or commercial data. This type of data could be important for researchers and scientists to better understand important health outcomes. However, GDPR restricts processing of this data without an explicit individual consent. | Q4-1: How willing would you be to share your commercial data with the government organisations? | --Definitely Yes  --Probably Yes  -- Probably No  --Definitely No |  |
|  |  | Q4-2: How willing would you be to share your commercial data with the private companies based in the UK? |  |  |
|  |  | Q4-3: How willing would you be to share your commercial data with universities and publicly funded research institutes in the UK? |  |  |
|  | Q5: As stated in the previous question, there are different types of commercial data that defined as special category data. Please rate your willingness to share your personal data collected through different methods. | Q5-1: How willing would you be to share your commercial data collected through your internet searches? | --Definitely Yes  --Probably Yes  -- Probably No  --Definitely No |  |
|  |  | Q5-2: How willing would you be to share your personal data collected through your buying habits collected using loyalty cards from high street retailers (e.g. Boots, Tesco, Nectar Card etc)? |  |  |
|  |  | Q5-3: How willing would you be to share your personal data collected through your smartphone applications that collect lifestyle and behaviour data? |  |  |
|  |  | Q5-4: How willing would you be to share your personal data collected through your social media posts (e.g. Facebook, Twitter)? |  |  |
|  |  | Q5-5: How willing would you be to share your personal data collected through your wearable devices (e.g. fitbit, garmin)? |  |  |
|  | Q6: Imagine that you were invited to a research study aiming to improve health outcomes using commercial data and you are eligible to take part. How willing would you be willing to provide your consent to take part in this research if you received an invitation through the following options. | Q6-1: A social media advertisement with a link to research study website associated with Scientists in universities and publicly funded research institutes. | --Definitely Yes  --Probably Yes  -- Probably No  --Definitely No |  |
|  |  | Q6-2: A newspaper advertisement with a link to take part in a research study associated with Scientists in universities and publicly funded research institutes. |  |  |
|  |  | Q6-3: A postal invitation letter from your health care provider. |  |  |
|  |  | Q6-4: A postal invitation from a high street retailer which you normally use for shopping. |  |  |
|  |  | Q6-5: A postal invitation from UK based scientists in universities and publicly funded research institutes. |  |  |
|  |  | Q6-6: A postal invitation from the UK government. |  |  |
|  |  | Q6-7: An email invitation from a high street retailer which you already use for shopping. |  |  |
|  |  | Q6-8: An email invitation from your healthcare provider. |  |  |
|  |  | Q6-9: An email invitation from a government institution. |  |  |
|  |  | Q6-10: An email invitation from Scientists in universities and publicly funded research institutes. |  |  |
|  |  | Q6-11: To what extent, do you think you would have control over your personal data if you take part in research based in an academic institution? |  |  |
|  |  | Q6-12: Most researchers are now encouraged to publish their anonymised datasets, i.e. complete dataset with personal identifiers are removed and participants cannot be identified, on a data repository so that new questions can be explored. Researchers will need to request additional consent from their participants to be able to publish their datasets. How willing would you be to provide permission for your anonymised data to be published in a data repository, like UK Data Archive and UK Data Service? |  |  |
| Trust and Privacy | Q7: Please indicate, to what extent, do you agree or disagree with the following statements. | Q7-1: Data privacy is important to me. | --Strongly agree  --Agree  --Neither agree nor disagree  --Disagree  --Strongly disagree | Jarvenpaa SL, Tractinsky N, Saarinen L (1999) Consumer Trust in an Internet Store: A Cross-Cultural Validation, Journal of Computer-Mediated Communication, 5(2)  Bhattacherjee A (2002) Individual Trust in Online Firms: Scale Development and Initial Test, Journal of management Information System, 19, 211-241 |
|  |  | Q7-2: I feel uncomfortable when other people have access to my personal data. |  |  |
|  |  | Q7-3: I trust research carried out by the government. |  |  |
|  |  | Q7-4: I trust research carried out by the commercial organisations. |  |  |
|  |  | Q7-5: I trust research carried out by academic institutions. |  |  |
|  |  | Q7-6: Scientists in universities and publicly funded research institutes have the skills and expertise to handle the public’s personal data in a way that protects their privacy. |  |  |
|  |  | Q7-7: Scientists in universities and publicly funded research institutes have access to the information and skills to handle the public’s personal data securely. |  |  |
|  |  | Q7-8: The way scientists in universities and publicly funded research institutes make the decisions to handle the public’s personal data is transparent. |  |  |
|  |  | Q7-9: The way scientists in universities and publicly funded research institutes make the decisions on how to handle the public’s personal data is fair. |  |  |
|  |  | Q7-10: Scientists in universities and publicly funded research institutes are fair in their user policies regarding the use of the public’s personal data. |  |  |
|  |  | Q7-11: Scientists in universities and publicly funded research institutes keep the public’s best interest in mind when handling their data. |  |  |
|  |  | Q7-12: Scientists in universities and publicly funded research institutes have the same opinions as me about handling the public's data. |  |  |
|  |  | Q7-13: In general, it would be risky to give my data to scientists in universities and publicly funded research institutes. |  |  |
|  |  | Q7-14: There would be too much uncertainty associated with giving my data to scientists in universities and publicly funded research institutes. |  |  |
|  |  | Q7-8: Providing scientists in universities and publicly funded research institutes with my data would involve many unexpected problems. |  |  |
|  |  | Q7-16: I would feel safe giving my data to scientists in universities and publicly funded research institutes. |  |  |
| Attitudes towards participating in health research using commercial data | Q8: Please rate to what extent you perceive the following concerns of participating in a health research using commercial data. | Q8-1: I would worry about the privacy of my data. | --Always  --Often  --Sometimes  --Rarely  --Never | Saskia C. Sanderson et.al)(2017): Public Attitudes toward Consent and Data Sharing in Biobank Research: A Large Multi-site Experimental Survey in the US  17-4: Seltzer, E., et.al (2019). Patients’ willingness to share digital health and non-health data for research: a cross-sectional study. *BMC medical informatics and decision making*, *19*(1), 87. |
|  |  | Q8-2: I would worry about the abuse of my data. |  |  |
|  |  | Q8-3: I would worry about the intention of the data collection. |  |  |
|  |  | Q8-4: I would worry organizations might use my information for purposes not stated in the consent or privacy policy. |  |  |
|  |  | Q8-5: I would worry my personal data will be shared to others without my consent. |  |  |
|  |  | Q8-6: I would worry someone would use my personal data to make money. |  |  |
|  | Q9: Please rate, to what extent you perceive the following benefits of participating in a health research using commercial data. | Q9-1: My participation could help future generations. | --Always  --Often  --Sometimes  --Rarely  --Never | Saskia C. Sanderson et.al)(2017): Public Attitudes toward Consent and Data Sharing in Biobank Research: A Large Multi-site Experimental Survey in the US |
|  |  | Q9-2: My participation could lead to better medical treatments. |  |  |
|  |  | Q9-3: My participation could improve the patients' health outcomes. |  |  |
|  |  | Q9-4: My participation could help my family |  |  |
|  |  | Q9-5: My participation could help me personally |  |  |
|  | Q10: Please indicate have any of the following events happened to you online. | Q10-1: An account of yours was accessed by someone you didn't give permission. | --Yes  --No  --Unsure | Seltzer, E., Goldshear, J., Guntuku, S.C. et al. Patients’ willingness to share digital health and non-health data for research: a cross-sectional study. BMC Med Inform Decis Mak 19, 157 (2019). https://doi.org/10.1186/s12911-019-0886-9 |
|  |  | Q10-2: The privacy of your personal information was violated. |  |  |
|  |  | Q10-3: Your reputation was negatively affected as a result of information posted online. |  |  |
|  |  | Q10-4: You had an unpleasant experience as a result of information you gave out online. |  |  |
|  |  | Q10-5: You were the victim of fraud and/or identity theft. |  |  |
| Demographics | Q11: Please indicate your age below. |  | --18-24  --25-34  --35-44  --45-54  --55-64  --65 and above |  |
|  | Q12: What is your gender |  | --Male  --Female  --Other |  |
|  | Q13: Please indicate your highest education level below. |  | -- Higher Education or professional/ vocational equivalents  -- A Levels or vocational level 3 equivalents  -- GCSE/ O Level grade A*-C or vocational level 2 and equivalents  -- Qualifications at vocational level 1 and below  -- Other qualifications: level unknown including foreign qualifications  -- No formal qualifications  --Prefer not to say |  |
|  | Q14: What is your current legal marital status? |  | --Single, never married  -- Married  -- A civil partner in a legally-  -- Legally separated  -- Divorced  -- Widowed  --Prefer not to say |  |
|  | Q15: Please specify your ethnicity. |  | --Black  --White  --Asian  --Mixed  --Other  --Prefer not to say |  |
|  | Q16: Where do you live? |  | -- London  -- East of England  -- South East  -- South West  -- West Midlands  -- East Midlands  -- Yorkshire and the Humber  -- North West  -- North East  -- Scotland  -- Wales  -Prefer not to say |  |
|  | Q17: Have you ever participated in any health-related research before? |  | --No  --Yes |  |

1. Kantar TNS UK. GDPR Awareness Index. 2018. Available at: <https://www.tnsglobal.co.uk/GDPR> [Accessed February 2022]
2. Bhattacherjee A. Individual trust in online firms: Scale development and initial test. Journal of management information systems. 2002 Jul 1;19(1):211-41.
3. Jarvenpaa SL, Tractinsky N, Saarinen L. Consumer trust in an Internet store: A cross-cultural validation. Journal of Computer-Mediated Communication. 1999 Dec 1;5(2):JCMC526.
4. Sanderson SC, Brothers KB, Mercaldo ND, Clayton EW, Antommaria AH, Aufox SA, Brilliant MH, Campos D, Carrell DS, Connolly J, Conway P. Public attitudes toward consent and data sharing in biobank research: a large multi-site experimental survey in the US. The American Journal of Human Genetics. 2017 Mar 2;100(3):414-27.
5. Seltzer, E., Goldshear, J., Guntuku, S.C. et al. Patients’ willingness to share digital health and non-health data for research: a cross-sectional study. BMC Med Inform Decis Mak 19, 157 (2019). <https://doi.org/10.1186/s12911-019-0886-9>

| **Supplementary Material Section 4: “General Data Protection Regulation : General Awareness** |
| --- |

| Supplementary Table 4: Participants’ expectations for what information is considered personal data | | | |
| --- | --- | --- | --- |
|  | Yes | No | I don’t know |
| Name | 1337 (87.2%) | 124 (8.1%) | 73 (4.8%) |
| Age | 1330 (86.7%) | 134 (8.7%) | 70 (4.6%) |
| Gender | 1301 (84.8%) | 158 (10.3%) | 75 (4.9%) |
| Marital Status | 1252 (81.6%) | 173 (11.3%) | 109 (7.1%) |
| Sexual Orientation | 1121 (73.1%) | 278 (18.1%) | 135 (8.8%) |
| Religion | 1067 (69.6%) | 306 (19.9%) | 161 (10.5%) |
| Home Address | 1281 (83.5%) | 164 (10.7%) | 89 (5.8%) |
| Criminal Record | 1100 (71.7%) | 265 (17.3%) | 169 (11.0%) |
| Email Address | 1237(80.6%) | 193 (12.6%) | 104 (6.8%) |
| Health/medical Records | 1136 (74.1%) | 263 (17.1%) | 135 (8.8%) |
| Tracking data about which websites I have visited | 916 (59.7%) | 383 (25.0%) | 235 (15.3%) |
| Device IDs which enable companies or organization to send information personally to my computer or smartphone. | 901(58.7%) | 353 (23.0%) | 280 (18.3%) |
| Information on products and services I have bought online. | 921 (60.0%) | 393 (25.6%) | 220 (14.3%) |
| Information on my interests based on things I have linked on social media. | 863 (56.3%) | 424 (27.6%) | 247 (16.1%) |
| Information on my location based on GPRS data from my smartphone. | 961 (62.6%) | 353 (23.0%) | 220 (14.3%) |

| Supplementary Table 5 Participants’ expectations for what is considered to be covered under GDPR | | | |
| --- | --- | --- | --- |
|  | Yes | No | I don’t know |
| Companies / organisations need to clearly ask for my permission to use my personal data. | 1369 (89.2%) | 63 (4.1%) | 102 (6.6%) |
| I should be able to ask companies / organisations to stop marketing to me directly. | 1322 (86.2%) | 98 (6.4%) | 114 (7.4%) |
| Companies / organisations need to guarantee that any data they hold on me is not passed onto any other companies / organisations without my consent. | 1334 (87.0%) | 80 (5.2%) | 120 (7.8%) |
| Companies / organisations must notify me immediately of any breach of my personal data (that is, if my data is accessed from anyone outside the organisation). | 1319 (86.0%) | 80 (5.2%) | 135 (8.8%) |
| I can ask companies / organisations to erase any data they hold about me. | 1243 (81.0%) | 85 (5.5%) | 206 (13.4%) |
| I can ask companies / organisations to tell me what data they hold about me. | 1285 (83.8%) | 101 (6.6%) | 148 (9.6%) |
| Companies / organisations need to use plain and simple language to explain how they want to use my data. | 1258 (82.0%) | 123 (8.0%) | 188 (12.3%) |
| Companies / organisations must ask me to opt into them using my personal data rather than expecting me to opt out. | 1223 (79.7%) | 123 (8.0%) | 188 (12.3%) |
| Companies / organisations should pay heavy fines to the government in the event of any data breach. | 1236(80.6%) | 98 (6.4%) | 200 (13.0%) |
| Companies / organisations should compensate me in the event of any data breach. | 1086 (70.8%) | 175 (11.4%) | 273 (17.8%) |
| Companies / organisations should pay me or offer me goods and services in return for using my personal data. | 919 (59.9%) | 319 (20.8%) | 296 (19.3%) |

**Supplementary Material Section 5: Ordered Logistic regression results on willingness to share based on invitation sources**

Supplementary Table 6a. Ordered logistic regression on sources of research invitation for health research (1/2)

|  | Postal invitation from healthcare provider | | | Email invitation from healthcare provider | | | Postal invitation from UK government | | | Postal invitation from UK base scientists in universities | | | Email invitation from scientists in universities | | |
| --- | --- | --- | --- | --- | --- | --- | --- | --- | --- | --- | --- | --- | --- | --- | --- |
|  | aOR | 95% CI | p-value | aOR | 95% CI | p-value | aOR | 95% CI | p-value | aOR | 95% CI | p-value | aOR | 95% CI | p-vaue |
| **Gender** |  |  |  |  |  |  |  |  |  |  |  |  |  |  |  |
| Male | Ref. |  |  | Ref. |  |  | Ref. |  |  | Ref. |  |  | Ref. |  |  |
| Female | 1.084 | 0.893 - 1.317 | 0.415 | 0.957 | 0.788 - 1.162 | 0.657 | 0.966 | 0.796 - 1.173 | 0.727 | 0.987 | 0.814 - 1.198 | 0.898 | 0.936 | 0.771 - 1.136 | 0.505 |
| **Marital status** |  |  |  |  |  |  |  |  |  |  |  |  |  |  |  |
| Single | Ref. |  |  | Ref. |  |  | Ref. |  |  | Ref. |  |  | Ref. |  |  |
| Married/legal partnership | 1.267 | 0.996 - 1.612 | 0.054 | 1.122 | 0.883 - 1.426 | 0.346 | 1.278 | 1.005 - 1.624* | 0.045 | 1.299 | 1.021 - 1.652* | 0.033 | 1.216 | 0.957 - 1.545 | 0.109 |
| Widowed/divorced/sep. | 1.234 | 0.870 - 1.750 | 0.238 | 1.267 | 0.892 - 1.799 | 0.187 | 1.466 | 1.033 - 2.080* | 0.032 | 1.529 | 1.076 - 2.172* | 0.018 | 1.362 | 0.959 - 1.934 | 0.085 |
| **Age** |  |  |  |  |  |  |  |  |  |  |  |  |  |  |  |
| 18-29 | Ref. |  |  | Ref. |  |  | Ref. |  |  | Ref. |  |  | Ref. |  |  |
| 30-39 | 1.132 | 0.810 - 1.583 | 0.468 | 0.922 | 0.660 - 1.288 | 0.634 | 0.933 | 0.667 - 1.306 | 0.687 | 0.998 | 0.711 - 1.400 | 0.991 | 0.991 | 0.706 - 1.391 | 0.957 |
| 40-49 | 0.768 | 0.543 - 1.086 | 0.135 | 0.852 | 0.603 - 1.205 | 0.366 | 0.655 | 0.463 - 0.927* | 0.017 | 0.775 | 0.547 - 1.099 | 0.153 | 0.833 | 0.588 - 1.180 | 0.304 |
| 50-59 | 1.059 | 0.740 - 1.514 | 0.755 | 0.883 | 0.619 - 1.261 | 0.495 | 0.777 | 0.543 - 1.110 | 0.165 | 0.676 | 0.472 - 0.969* | 0.033 | 0.687 | 0.480 - 0.983* | 0.040 |
| 60-69 | 1.415 | 0.964 - 2.077 | 0.076 | 1.050 | 0.718 - 1.536 | 0.800 | 0.804 | 0.549 - 1.177 | 2.261 | 0.753 | 0.513 - 1.106 | 0.148 | 0.698 | 0.476 - 1.024 | 0.066 |
| 70+ | 1.601 | 1.094 - 2.344* | 0.015 | 1.294 | 0.885 - 1.892 | 0.183 | 1.225 | 0.837 - 1.793 | 0.296 | 0.830 | 0.567 - 1.216 | 0.340 | 0.822 | 0.562 - 1.202 | 0.311 |
| **Ethnicity** |  |  |  |  |  |  |  |  |  |  |  |  |  |  |  |
| Black | Ref. |  |  | Ref. |  |  | Ref. |  |  | Ref. |  |  | Ref. |  |  |
| White | 0.746 | 0.409 - 1.361 | 0.340 | 0.615 | 0.337 - 1.121 | 0.112 | 0.496 | 0.272 - 0.907* | 0.023 | 0.581 | 0.315 - 1.071 | 0.082 | 0.593 | 0.327 - 1.078 | 0.087 |
| Asian | 0.691 | 0.352 - 1.356 | 0.283 | 0.601 | 0.306 - 1.179 | 0.139 | 0.510 | 0.260 - 1.002 | 0.051 | 0.485 | 0.245 - 0.960* | 0.038 | 0.548 | 0.280 - 1.072 | 0.079 |
| Mixed | 0.862 | 0.353 - 2.109 | 0.745 | 0.880 | 0.355 - 2.184 | 0.783 | 0.586 | 0.242 - 1.419 | 0.236 | 0.374 | 0.151 - 0.929* | 0.034 | 0.497 | 0.198 - 1.250 | 0.137 |
| Other | 0.744 | 0.172 - 3.212 | 0.692 | 0.649 | 0.149 - 2.824 | 0.565 | 0.462 | 0.127 - 1.680 | 0.241 | 1.467 | 0.385 - 5.598 | 0.575 | 1.126 | 0.311 - 4.073 | 0.857 |
| **Education** |  |  |  |  |  |  |  |  |  |  |  |  |  |  |  |
| Below degree | Ref. |  |  | Ref. |  |  | Ref. |  |  | Ref. |  |  | Ref. |  |  |
| Degree or above | 1.107 | 0.907 - 1.351 | 0.316 | 0.993 | 0.814 - 1.212 | 0.946 | 1.057 | 0.866 - 1.290 | 0.589 | 1.195 | 0.978 - 1.460 | 0.082 | 1.243 | 1.018 - 1.518* | 0.033 |
| **Living area** |  |  |  |  |  |  |  |  |  |  |  |  |  |  |  |
| London | Ref. |  |  | Ref. |  |  | Ref. |  |  | Ref. |  |  | Ref. |  |  |
| East | 1.493 | 0.998 - 2.232 | 0.051 | 1.296 | 0.867 - 1.937 | 0.206 | 1.745 | 1.167 - 2.608** | 0.007 | 1.316 | 0.884 - 1.961 | 0.177 | 1.121 | 0.753 - 1.669 | 0.575 |
| South East | 1.189 | 0.825 - 1.714 | 0.352 | 0.970 | 0.675 - 1.393 | 0.868 | 1.210 | 0.843 - 1.736 | 0.301 | 1.181 | 0.822 - 1.698 | 0.369 | 0.961 | 0.669 - 1.380 | 0.829 |
| South West | 1.405 | 0.928 - 2.128 | 0.108 | 1.434 | 0.948 - 2.167 | 0.088 | 1.479 | 0.979 - 2.237 | 0.063 | 1.294 | 0.858 - 1.951 | 0.219 | 1.108 | 0.737 - 1.666 | 0.622 |
| West & East Midlands | 1.117 | 0.786 - 1.588 | 0.537 | 1.199 | 0.846 - 1.698 | 0.308 | 1.359 | 0.957 - 1.929 | 0.086 | 0.953 | 0.673 - 1.350 | 0.786 | 0.819 | 0.577 - 1.162 | 0.262 |
| Yorkshire and Humber | 1.203 | 0.830 - 1.745 | 0.329 | 1.286 | 0.885 - 1.868 | 0.187 | 1.171 | 0.810 - 1.693 | 0.400 | 1.037 | 0.712 - 1.510 | 0.849 | 0.869 | 0.599 - 1.262 | 0.462 |
| North West | 1.042 | 0.717 - 1.514 | 0.830 | 1.010 | 0.700 - 1.458 | 0.957 | 1.069 | 0.736 - 1.552 | 0.727 | 1.008 | 0.697 - 1.458 | 0.965 | 0.954 | 0.659 - 1.383 | 0.805 |
| Scotland | 0.860 | 0.571 - 1.297 | 0.472 | 0.885 | 0.589 - 1.329 | 0.555 | 0.844 | 0.558 - 1.277 | 0.422 | 0.956 | 0.635 - 1.440 | 0.831 | 0.903 | 0.596 - 1.368 | 0.630 |
| Wales | 0.853 | 0.503 - 1.449 | 0.557 | 0.931 | 0.551 - 1.571 | 0.788 | 0.890 | 0.528 - 1.500 | 0.662 | 1.027 | 0.609 - 1.732 | 0.921 | 0.877 | 0.521 - 1.477 | 0.622 |
| **GDPR awareness** |  |  |  |  |  |  |  |  |  |  |  |  |  |  |  |
| None | Ref. |  |  | Ref. |  |  | Ref. |  |  | Ref. |  |  | Ref. |  |  |
| Heard, but know not much | 1.285 | 0.920 - 1.795 | 0.141 | 1.267 | 0.906 - 1.772 | 0.167 | 1.595 | 1.143 - 2.225** | 0..006 | 1.810 | 1.300 - 2.520** | <0.001 | 1.601 | 1.151 - 2.227** | 0.005 |
| Heard, and know little | 1.490 | 1.082 - 2.052* | 0.015 | 1.624 | 1.180 - 2.236** | 0.003 | 1.808 | 1.314 - 2.488** | <0.001 | 1.928 | 1.406 - 2.643** | <0.001 | 1.745 | 1.273 - 2.391** | 0.001 |
| Heard, and know a lot | 2.143 | 1.468 - 3.129** | <0.001 | 2.065 | 1.418 - 3.007** | <0.001 | 2.325 | 1.598 - 3.381** | <0.001 | 2.776 | 1.905 - 4.046** | <0.001 | 2.469 | 1.701 - 3.585** | <0.001 |
| **Participated before** |  |  |  |  |  |  |  |  |  |  |  |  |  |  |  |
| Yes | Ref. |  |  | Ref. |  |  | Ref. |  |  | Ref. |  |  | Ref. |  |  |
| No | 0.979 | 0.783 - 1.223 | 0.849 | 0.818 | 0.655 - 1.023 | 0.078 | 0.801 | 0.642 - 0.999* | 0.049 | 0.767 | 0.615 - 0.958* | 0.019 | 0.670 | 0.536 - 0.837** | <0.001 |
| *R^2^* | 0.0164 |  |  | 0.0123 |  |  | 0.0195 |  |  | 0.0175 |  |  | 0.0181 |  |  |
| N | 1,494 |  |  | 1,494 |  |  | 1,494 |  |  | 1,494 |  |  | 1,494 |  |  |

* *p*<0.05; ** *p*<0.01

Table 6b. Ordered logistic regression on sources of research invitation for health research (2/2)

|  | Email invitation from scientist from a government institution | | | Social media advertisement with link to study website associated | | | Email invitation from a high street retailer you use | | | A newspaper advertisement with a link to take part in study | | | Postal invitation from a high street retailer you use | | |
| --- | --- | --- | --- | --- | --- | --- | --- | --- | --- | --- | --- | --- | --- | --- | --- |
|  | aOR | 95% CI | p-value | aOR | 95% CI | p-value | aOR | 95% CI | p-value | aOR | 95% CI | p-value | aOR | 95% CI | p-value |
| **Gender** |  |  |  |  |  |  |  |  |  |  |  |  |  |  |  |
| Male | Ref. |  |  | Ref. |  |  | Ref. |  |  | Ref. |  |  | Ref. |  |  |
| Female | 0.911 | 0.752 - 1.104 | 0.343 | 0.942 | 0.779 - 1.139 | 0.540 | 0.770 | 0.636 - 0.932** | 0.007 | 0.860 | 0.711 - 1.040 | 0.119 | 0.815 | 0.673 - 0.988* | 0.037 |
| **Marital status** |  |  |  |  |  |  |  |  |  |  |  |  |  |  |  |
| Single | Ref. |  |  | Ref. |  |  | Ref. |  |  | Ref. |  |  | Ref. |  |  |
| Married/legal partnership | 1.165 | 0.920 - 1.476 | 0.206 | 1.228 | 0.971 - 1.554 | 0.087 | 1.268 | 1.000 - 1.609 | 0.050 | 1.261 | 0.996 - 1.597 | 0.054 | 1.441 | 1.136 - 1.829** | 0.003 |
| Widowed/divorced/sep. | 1.159 | 0.820 - 1.638 | 0.403 | 1.211 | 0.862 - 1.702 | 0.270 | 1.279 | 0.906 - 1.806 | 0.161 | 1.203 | 0.858 - 1.687 | 0.283 | 1.390 | 0.986 - 1.959 | 0.060 |
| **Age** |  |  |  |  |  |  |  |  |  |  |  |  |  |  |  |
| 18-29 | Ref. |  |  | Ref. |  |  | Ref. |  |  | Ref. |  |  | Ref. |  |  |
| 30-39 | 1.032 | 0.740 - 1.440 | 0.853 | 0.831 | 0.598 - 1.154 | 0.269 | 0.871 | 0.624 - 1.216 | 0.417 | 0.847 | 0.608 - 1.179 | 0.325 | 0.922 | 0.661 - 1.286 | 0.632 |
| 40-49 | 0.891 | 0.632 - 1.255 | 0.509 | 0.549 | 0.389 - 0.775** | 0.001 | 0.574 | 0.405 - 0.812** | 0.002 | 0.542 | 0.383 - 0.766** | 0.001 | 0.583 | 0.411 - 0.827** | 0.002 |
| 50-59 | 0.827 | 0.580 - 1.177 | 0.291 | 0.352 | 0.247 - 0.502** | <0.001 | 0.379 | 0.266 - 0.541** | <0.001 | 0.412 | 0.289 - 0.587** | <0.001 | 0.427 | 0.299 - 0.610** | <0.001 |
| 60-69 | 0.846 | 0.580 - 1.235 | 0.387 | 0.233 | 0.159 - 0.342** | <0.001 | 0.285 | 0.195 - 0.419** | <0.001 | 0.353 | 0.241 - 0.515** | <0.001 | 0.337 | 0.230 - 0.495** | <0.001 |
| 70+ | 1.095 | 0.752 - 1.594 | 0.635 | 0.300 | 0.206 - 0.437** | <0.001 | 0.259 | 0.177 - 0.378** | <0.001 | 0.333 | 0.229 - 0.483** | <0.001 | 0.278 | 0.191 - 0.406** | <0.001 |
| **Ethnicity** |  |  |  |  |  |  |  |  |  |  |  |  |  |  |  |
| Black | Ref. |  |  | Ref. |  |  | Ref. |  |  | Ref. |  |  | Ref. |  |  |
| White | 0.641 | 0.355 - 1.155 | 0.139 | 0.694 | 0.384 - 1.253 | 0.226 | 0.519 | 0.284 - 0.948* | 0.033 | 0.472 | 0.259 - 0.859* | 0.014 | 0.540 | 0.302 - 0.965* | 0.037 |
| Asian | 0.663 | 0.342 - 1.286 | 0.224 | 0.823 | 0.426 - 1.593 | 0.564 | 0.695 | 0.354 - 1.365 | 0.291 | 0.486 | 0.249 - 0.948* | 0.034 | 0.609 | 0.315 - 1.174 | 0.139 |
| Mixed | 1.020 | 0.418 - 2.490 | 0.965 | 0.763 | 0.322 - 1.810 | 0.540 | 0.541 | 0.222 - 1.315 | 0.175 | 0.542 | 0.218 - 1.346 | 0.187 | 0.393 | 0.168 - 0.923* | 0.032 |
| Other | 0.580 | 0.154 - 2.187 | 0.422 | 0.918 | 0.242 - 3.478 | 0.900 | 0.717 | 0.163 - 3.162 | 0.660 | 0.733 | 0.194 - 2.772 | 0.648 | 0.580 | 0.145 - 2.330 | 0.443 |
| **Education** |  |  |  |  |  |  |  |  |  |  |  |  |  |  |  |
| Below degree | Ref. |  |  | Ref. |  |  | Ref. |  |  | Ref. |  |  | Ref. |  |  |
| Degree or above | 1.004 | 0.824 - 1.224 | 0.966 | 0.961 | 0.791 - 1.167 | 0.686 | 0.809 | 0.664 - 0.985* | 0.035 | 1.125 | 0.924 - 1.370 | 0.240 | 0.844 | 0.693 - 1.028 | 0.092 |
| **Living area** |  |  |  |  |  |  |  |  |  |  |  |  |  |  |  |
| London | Ref. |  |  | Ref. |  |  | Ref. |  |  | Ref. |  |  | Ref. |  |  |
| East | 1.395 | 0.938 - 2.075 | 0.100 | 1.614 | 1.088 - 2.394* | 0.017 | 1.261 | 0.849 - 1.874 | 0.251 | 1.169 | 0.789 - 1.731 | 0.437 | 0.941 | 0.631 - 1.401 | 0.764 |
| South East | 1.245 | 0.869 - 1.785 | 0.232 | 1.100 | 0.770 - 1.572 | 0.601 | 1.098 | 0.767 - 1.571 | 0.611 | 1.006 | 0.705 - 1.435 | 0.974 | 1.054 | 0.735 - 1.511 | 0.777 |
| South West | 1.492 | 0.992 - 2.245 | 0.055 | 1.194 | 0.793 - 1.798 | 0.397 | 1.122 | 0.750 - 1.680 | 0.575 | 1.039 | 0.693 - 1.558 | 0.854 | 1.155 | 0.772 - 1.727 | 0.484 |
| West & East Midlands | 1.424 | 1.006 - 2.015* | 0.046 | 1.248 | 0.888 - 1.754 | 0.201 | 1.118 | 0.790 - 1.580 | 0.529 | 0.946 | 0.672 - 1.333 | 0.753 | 1.019 | 0.721 - 1.440 | 0.917 |
| Yorkshire and Humber | 1.116 | 0.774 - 1.609 | 0.558 | 1.124 | 0.786 - 1.607 | 0.523 | 0.836 | 0.580 - 1.203 | 0.335 | 0.895 | 0.624 - 1.285 | 0.549 | 0.880 | 0.608 - 1.273 | 0.498 |
| North West | 1.099 | 0.761 - 1.585 | 0.615 | 1.314 | 0.917 - 1.882 | 0.137 | 1.106 | 0.765 - 1.598 | 0.592 | 1.218 | 0.846 - 1.752 | 0.289 | 0.997 | 0.690 - 1.441 | 0.988 |
| Scotland | 0.928 | 0.618 - 1.394 | 0.718 | 0.887 | 0.591 - 1.330 | 0.561 | 1.015 | 0.677 - 1.521 | 0.943 | 0.948 | 0.628 - 1.433 | 0.801 | 0.682 | 0.454 - 1.024 | 0.065 |
| Wales | 1.009 | 0.599 - 1.699 | 0.973 | 1.314 | 0.780 - 2.212 | 0.305 | 1.058 | 0.624 - 1.796 | 0.834 | 0.843 | 0.503 - 1.411 | 0.515 | 0.701 | 0.415 - 1.186 | 0.185 |
| **GDPR awareness** |  |  |  |  |  |  |  |  |  |  |  |  |  |  |  |
| None | Ref. |  |  | Ref. |  |  | Ref. |  |  | Ref. |  |  | Ref. |  |  |
| Heard, but know not much | 1.342 | 0.965 - 1.867 | 0.080 | 1.302 | 0.942 - 1.799 | 0.110 | 1.346 | 0.968 - 1.872 | 0.077 | 1.207 | 0.871 - 1.672 | 0.258 | 1.489 | 1.072 - 2.067* | 0.017 |
| Heard, and know little | 1.628 | 1.187 - 2.235** | 0.003 | 1.080 | 0.793 - 1.472 | 0.624 | 1.341 | 0.980 - 1.836 | 0.067 | 1.221 | 0.893 - 1.668 | 0.210 | 1.299 | 0.951 - 1.776 | 0.202 |
| Heard, and know a lot | 1.996 | 1.378 - 2.893** | <0.001 | 1.612 | 1.120 - 2.320* | 0.010 | 1.744 | 1.203 - 2.528** | 0.003 | 1.804 | 1.245 - 2.612** | 0.002 | 1.793 | 1.238 - 2.595** | 0.002 |
| **Participated before** |  |  |  |  |  |  |  |  |  |  |  |  |  |  |  |
| Yes | Ref. |  |  | Ref. |  |  | Ref. |  |  | Ref. |  |  | Ref. |  |  |
| No | 0.757 | 0.607 - 0.944* | 0.014 | 0.819 | 0.660 - 1.018 | 0.071 | 0.873 | 0.702 - 1.087 | 0.225 | 0.911 | 0.734 - 1.132 | 0.400 | 1.063 | 0.855 - 1.321 | 0.582 |
| *R^2^* | 0.0122 |  |  | 0.0346 |  |  | 0.0373 |  |  | 0.0286 |  |  | 0.0331 |  |  |
| N | 1,494 |  |  | 1,494 |  |  | 1,494 |  |  | 1,494 |  |  | 1,494 |  |  |

* *p*<0.05; ** *p*<0.01

| Table 3· Psychological predictors of willingness to share commercial data for health research with different organisations· (N=1534) | | | | | | | | | |
| --- | --- | --- | --- | --- | --- | --- | --- | --- | --- |
|  | Government Institutes | | | Private Institutes | | | Academic Institutes | | |
|  | aOR | 95% CI | p-value | aOR | 95% CI | p-value | aOR | 95% CI | p-value |
| Trust in organisations | 2.499 | 2.228 - 2.802** | <0.001 | 2·513 | 2.221 – 2.842** | <0.001 | 2.283 | 2.011 – 2.590** | <0.001 |
| Worry about data misuse | 0.940 | 0.918 - 0.961** | <0.001 | 0·951 | 0.930 – 0.973** | <0.001 | 0.947 | 0.926 – 0.969** | <0.001 |
| Perceived risk of data donation | 1.041 | 0.997 - 1.086 | 0.067 | 1·042 | 0.997 – 1.089 | 0.071 | 1.016 | 0.974 – 1.060 | 0.452 |
| Perceived importance of privacy | 0.909 | 0.833 - 0.992* | 0.031 | 0·833 | 0.763 – 0.909** | <0.001 | 0.869 | 0.797 – 0.948** | 0.002 |
| Perceived benefits of sharing data and participation | 1.111 | 1.083 - 1.140** | <0.001 | 1·081 | 1.054 – 1.109** | <0.001 | 1.116 | 1.087 – 1.146** | <0.001 |
| Age |  |  |  |  |  |  |  |  |  |
| 18-29 | Ref. |  |  | Ref. |  |  | Ref. |  |  |
| 30-39 | 1.055 | 0.742 - 1.500 | 0.766 | 1.137 | 0.803 - 1.610 | 0.471 | 1.061 | 0.747 - 1.505 | 0.714 |
| 40-49 | 0.679 | 0.472 - 0.975* | 0.036 | 0.776 | 0.541 - 1.112 | 0.167 | 0.744 | 0.519 - 1.068 | 0.109 |
| 50-59 | 0.706 | 0.485 - 1.027 | 0.069 | 0.561 | 0.386 - 0.815** | 0.002 | 0.666 | 0.460 - 0.965* | 0.032 |
| 60-69 | 0.758 | 0.505 - 1.137 | 0.180 | 0.600 | 0.400 - 0.899* | 0.013 | 0.781 | 0.523 - 1.166 | 0.227 |
| 70+ | 0.924 | 0.620 - 1.377 | 0.697 | 0.564 | 0.379 - 0.839** | 0.005 | 0.761 | 0.512 - 1.130 | 0.176 |
| Ethnicity |  |  |  |  |  |  |  |  |  |
| Black | Ref. |  |  | Ref. |  |  | Ref. |  |  |
| White | 0.586 | 0.311 - 1.104 | 0.098 | 0.529 | 0.276 - 1.012 | 0.054 | 0.909 | 0.487 - 1.697 | 0.764 |
| Asian | 0.443 | 0.218 - 0.903* | 0.025 | 0.331 | 0.160 - 0.683** | 0.003 | 0.575 | 0.285 - 1.162 | 0.123 |
| Mixed | 0.426 | 0.166 - 1.093 | 0.076 | 0.532 | 0.209 - 1.359 | 0.187 | 0.479 | 0.192 - 1.192 | 0.113 |
| Other | 0.447 | 0.104 - 1.920 | 0.279 | 0.355 | 0.080 - 1.575 | 0.173 | 0.402 | 0.088 - 1.842 | 0.240 |
| Education |  |  |  |  |  |  |  |  |  |
| Below degree | Ref. |  |  | Ref. |  |  | Ref. |  |  |
| Degree or above | 1.207 | 0.979 - 1.488 | 0.077 | 0.803 | 0.653 - 0.988* | 0.038 | 1.068 | 0.867 - 1.316 | 0.536 |
| GDPR awareness |  |  |  |  |  |  |  |  |  |
| None | Ref. |  |  | Ref. |  |  | Ref. |  |  |
| Heard, but know not much | 1.184 | 0.837 - 1.675 | 0.340 | 1.419 | 1.002 - 2.010* | 0.049 | 1.339 | 0.949 - 1.888 | 0.096 |
| Heard, and know little | 1.104 | 0.792 - 1.537 | 0.560 | 1.543 | 1.106 - 2.153* | 0.011 | 1.321 | 0.952 - 1.834 | 0.095 |
| Heard, and know a lot | 1.495 | 1.010 - 2.214* | 0.045 | 1.589 | 1.072 - 2.357* | 0.021 | 1.546 | 1.048 - 2.281* | 0.028 |
| Participated before |  |  |  |  |  |  |  |  |  |
| No | Ref. |  |  | Ref. |  |  | Ref. |  |  |
| Yes | 0.999 | 0.793 - 1.258 | 0.992 | 0.896 | 0.712 - 1.127 | 0.347 | 0.857 | 0.681 - 1.079 | 0.190 |
| Location |  |  |  |  |  |  |  |  |  |
| London | Ref. |  |  | Ref. |  |  | Ref. |  |  |
| East | 1.541 | 1.013 - 2.345* | 0.043 | 0.986 | 0.653 - 1.488 | 0.946 | 1.159 | 0.768 - 1.750 | 0.481 |
| South East | 1.055 | 0.723 - 1.539 | 0.783 | 1.034 | 0.710 - 1.505 | 0.863 | 1.001 | 0.686 - 1.460 | 0.996 |
| South West | 1.288 | 0.838 - 1.979 | 0.249 | 0.987 | 0.645 - 1.510 | 0.951 | 1.189 | 0.774 - 1.825 | 0.429 |
| West & East Midlands | 1.430 | 0.992 - 2.062 | 0.055 | 1.009 | 0.702 - 1.451 | 0.960 | 1.185 | 0.823 - 1.707 | 0.361 |
| Yorkshire and Humber | 1.044 | 0.711 - 1.534 | 0.826 | 0.885 | 0.604 - 1.297 | 0.532 | 0.831 | 0.567 - 1.217 | 0.342 |
| North West | 1.278 | 0.867 - 1.884 | 0.215 | 1.023 | 0.695 - 1.507 | 0.906 | 1.178 | 0.804 - 1.724 | 0.401 |
| Scotland | 1.285 | 0.833 - 1.983 | 0.257 | 1.257 | 0.818 - 1.931 | 0.297 | 1.202 | 0.782 - 1.846 | 0.401 |
| Wales | 0.757 | 0.427 - 1.342 | 0.341 | 1.249 | 0.717 - 2.174 | 0.432 | 0.928 | 0.531 - 1.620 | 0.792 |
| Gender |  |  |  |  |  |  |  |  |  |
| Male | Ref. |  |  | Ref. |  |  | Ref. |  |  |
| Female | 0.701 | 0.572 - 0.858** | <0.001 | 0.741 | 0.606 - 0.905** | 0.003 | 0.836 | 0.684 - 1.022 | 0.080 |
| Marital status |  |  |  |  |  |  |  |  |  |
| Single | Ref. |  |  | Ref. |  |  | Ref. |  |  |
| Married/legal partnership | 1.231 | 0.957 - 1.582 | 0.105 | 1.109 | 0.865 - 1.421 | 0.415 | 1.096 | 0.854 - 1.406 | 0.473 |
| Widowed/divorced/seperated | 1.375 | 0.960 - 1.969 | 0.082 | 1.236 | 0.867 - 1.763 | 0.241 | 1.229 | 0.861 - 1.754 | 0.257 |
| *R^2^* | 0.2219 |  |  | 0.1597 |  |  | 0.1419 |  |  |
| N | 1494 |  |  | 1494 |  |  | 1494 |  |  |

* *p*<0.05; ** *p*<0.01

| Table 5. Psychological predictors of willingness to share different types of commercial data for health research with academic institutions (n=1534) | | | | | | | | | | | | | | | |
| --- | --- | --- | --- | --- | --- | --- | --- | --- | --- | --- | --- | --- | --- | --- | --- |
|  | Internet search  data | | | Loyalty card  data | | | Smart Phone data | | | Social Media data | | | Wearable devices data | | |
|  | aOR | 95% CI | P- value | aOR | 95% CI | p-value | aOR | 95% CI | p-value | aOR | 95% CI | p-value | aOR | 95% CI | p-value |
| Trust in data practices in academic institutions | 1.097 | 1.078 – 1.117** | <0.001 | 1.103 | 1.083 – 1.123** | <0.001 | 1.097 | 1.077 – 1.117** | <0.001 | 1.087 | 1.067 – 1.107** | <0.001 | 1.075 | 1.056 – 1.095** | <0.001 |
| Perceived importance of privacy | 0.682 | 0.625 – 0.744** | <0.001 | 0.707 | 0.648 - 0·.72** | <0.001 | 0.685 | 0.628 – 0.748** | <0.001 | 0.716 | 0.656 – 0.781** | <0.001 | 0.732 | 0.670 – 0.798** | <0.001 |
| Worry about data misuse | 0.960 | 0.938 – 0.982** | <0.001 | 0.975 | 0.953 – 0.997* | 0.029 | 0.960 | 0.938 – 0.982** | <0.001 | 0.963 | 0.941 – 0.985** | 0.001 | 0.940 | 0.919 – 0.962** | <0.001 |
| Perceived benefit in data sharing and research participation | 1.057 | 1.029 – 1.086** | <0.001 | 1.102 | 1.072 – 1.132** | <0.001 | 1.070 | 1.040 – 1.100** | <0.001 | 1.049 | 1.020 – 1.078** | 0.001 | 1.086 | 1.056 – 1.116** | <0.001 |
| Perceived risks of data donation | 1.222 | 1.169 – 1.276** | <0.001 | 1.114 | 1.067 – 1.163** | <0.001 | 1.224 | 1.171 – 1.279** | <0.001 | 1.208 | 1.156 – 1.263** | <0.001 | 1.232 | 1.179 – 1.287** | <0.001 |
| Age |  |  |  |  |  |  |  |  |  |  |  |  |  |  |  |
| 18-29 | Ref. |  |  | Ref. |  |  | Ref. |  |  | Ref. |  |  | Ref. |  |  |
| 30-39 | 1.361 | 0.969 - 1.912 | 0.076 | 0.926 | 0.654 - 1.312 | 0.666 | 0.946 | 0.674 - 1.329 | 0.751 | 1.054 | 0.756 - 1.470 | 0.756 | 1.265 | 0.905 - 1.769 | 0.168 |
| 40-49 | 0.938 | 0.660 - 1.333 | 0.721 | 0.793 | 0.554 - 1.133 | 0.203 | 0.785 | 0.552 - 1.117 | 0.179 | 0.613 | 0.432 - 0.869** | 0.006 | 0.622 | 0.439 - 0.881** | 0.008 |
| 50-59 | 0.550 | 0.380 - 0.794** | 0.001 | 0.665 | 0.459 - 0.963* | 0.031 | 0.469 | 0.323 - 0.682** | <0.001 | 0.392 | 0.272 - 0.567** | <0.001 | 0.357 | 0.246 - 0.516** | <0.001 |
| 60-69 | 0.554 | 0.373 - 0.825** | 0.004 | 0.613 | 0.411 - 0.915* | 0.017 | 0.365 | 0.244 - 0.546** | <0.001 | 0.272 | 0.182 - 0.408** | <0.001 | 0.281 | 0.188 - 0.421** | <0.001 |
| 70+ | 0.471 | 0.318 - 0.696** | <0.001 | 0.521 | 0.351 - 0.772** | 0.001 | 0.266 | 0.178 - 0.397** | <0.001 | 0.232 | 0.156 - 0.344** | <0.001 | 0.206 | 0.138 - 0.308** | <0.001 |
| Ethnicity |  |  |  |  |  |  |  |  |  |  |  |  |  |  |  |
| Black | Ref. |  |  | Ref. |  |  | Ref. |  |  | Ref. |  |  | Ref. |  |  |
| White | 0.820 | 0.448 - 1.502 | 0.521 | 0.784 | 0.415 - 1.482 | 0.453 | 0.773 | 0.430 - 1.389 | 0.388 | 0.516 | 0.287 - 0.929* | 0.027 | 0.628 | 0.342 - 1.151 | 0.132 |
| Asian | 0.897 | 0.454 - 1.773 | 0.755 | 0.667 | 0.329 - 1.349 | 0.260 | 0.699 | 0.362 - 1.352 | 0.288 | 0.492 | 0.254 - 0.954* | 0.036 | 0.598 | 0.303 - 1.180 | 0.138 |
| Mixed | 0.796 | 0.334 - 1.900 | 0.608 | 1.066 | 0.429 - 2.651 | 0.890 | 0.853 | 0.354 - 2.057 | 0.724 | 0.503 | 0.209 - 1.214 | 0.127 | 0.679 | 0.280 - 1.646 | 0.392 |
| Other | 0.715 | 0.192 - 2.666 | 0.617 | 0.945 | 0.228 - 3.906 | 0.937 | 0.985 | 0.250 - 3.887 | 0.983 | 0.402 | 0.109 - 1.480 | 0.170 | 0.273 | 0.069 - 1.079 | 0.064 |
| Education |  |  |  |  |  |  |  |  |  |  |  |  |  |  |  |
| Below degree | Ref. |  |  | Ref. |  |  | Ref. |  |  | Ref. |  |  | Ref. |  |  |
| Degree or above | 0.877 | 0.715 - 1.077 | 0.210 | 0.743 | 0.604 - 0.913** | 0.005 | 0.896 | 0.728 - 1.101 | 0.296 | 0.994 | 0.810 - 1.222 | 0.958 | 1.007 | 0.819 - 1.238 | 0.949 |
| GDPR awareness |  |  |  |  |  |  |  |  |  |  |  |  |  |  |  |
| None | Ref. |  |  | Ref. |  |  | Ref. |  |  | Ref. |  |  | Ref. |  |  |
| Heard, but know not much | 0.948 | 0.673 - 1.336 | 0.760 | 1.179 | 0.835 - 1.666 | 0.349 | 0.849 | 0.600 - 1.201 | 0.355 | 1.259 | 0.891 - 1.780 | 0.191 | 1.004 | 0.704 - 1.430 | 0.984 |
| Heard, and know little | 0.829 | 0.596 - 1.152 | 0.263 | 1.152 | 0.827 - 1.605 | 0.402 | 0.695 | 0.497 - 0.970* | 0.033 | 0.964 | 0.690 - 1.345 | 0.827 | 0.910 | 0.647 - 1.280 | 0.588 |
| Heard, and know a lot | 0.845 | 0.574 - 1.245 | 0.395 | 1.287 | 0.870 - 1.902 | 0.206 | 0.922 | 0.623 - 1.365 | 0.686 | 0.820 | 0.555 - 1.214 | 0.322 | 1.015 | 0.683 - 1.510 | 0.940 |
| Participated before |  |  |  |  |  |  |  |  |  |  |  |  |  |  |  |
| No | Ref. |  |  | Ref. |  |  | Ref. |  |  | Ref. |  |  | Ref. |  |  |
| Yes | 0.937 | 0.748 - 1.172 | 0.567 | 1.051 | 0.839 - 1.317 | 0.666 | 0.901 | 0.719 - 1.128 | 0.363 | 0.916 | 0.731 - 1.148 | 0.448 | 0.996 | 0.794 - 1.250 | 0.974 |
| Location |  |  |  |  |  |  |  |  |  |  |  |  |  |  |  |
| London | Ref. |  |  | Ref. |  |  | Ref. |  |  | Ref. |  |  | Ref. |  |  |
| East | 1.453 | 0.965 - 2.188 | 0.074 | 1.090 | 0.723 - 1.641 | 0.681 | 1.169 | 0.774 - 1.766 | 0.458 | 1.571 | 1.045 - 2.362* | 0.030 | 1.223 | 0.814 - 1.837 | 0.333 |
| South East | 0.793 | 0.547 - 1.150 | 0.221 | 0.903 | 0.623 - 1.310 | 0.591 | 0.835 | 0.575 - 1.213 | 0.344 | 0.835 | 0.576 - 1.213 | 0.344 | 0.849 | 0.585 - 1.231 | 0.388 |
| South West | 0.992 | 0.650 - 1.516 | 0.971 | 1.142 | 0.749 - 1.740 | 0.538 | 1.122 | 0.736 - 1.711 | 0.593 | 1.133 | 0.743 - 1.728 | 0.563 | 1.061 | 0.695 - 1.620 | 0.783 |
| West & East Midlands | 1.364 | 0.954 - 1.952 | 0.089 | 1.478 | 1.027 - 2.127* | 0.035 | 1.350 | 0.943 - 1.933 | 0.101 | 1.106 | 0.771 - 1.585 | 0.585 | 1.242 | 0.866 - 1.781 | 0.238 |
| Yorkshire and Humber | 0.992 | 0.679 - 1.450 | 0.968 | 0.821 | 0.562 - 1.201 | 0.310 | 0.885 | 0.605 - 1.296 | 0.532 | 1.201 | 0.822 - 1.755 | 0.343 | 1.091 | 0.746 - 1.596 | 0.653 |
| North West | 1.043 | 0.714 - 1.524 | 0.827 | 0.873 | 0.597 - 1.277 | 0.484 | 1.069 | 0.731 - 1.564 | 0.730 | 1.232 | 0.842 - 1.803 | 0.284 | 1.011 | 0.690 - 1.480 | 0.956 |
| Scotland | 1.023 | 0.667 - 1.570 | 0.916 | 0.852 | 0.557 - 1.303 | 0.459 | 1.084 | 0.708 - 1.660 | 0.711 | 1.068 | 0.697 - 1.637 | 0.764 | 0.905 | 0.588 - 1.393 | 0.651 |
| Wales | 1.074 | 0.614 - 1.880 | 0.801 | 0.982 | 0.572 - 1.686 | 0.947 | 1.084 | 0.624 - 1.884 | 0.774 | 1.257 | 0.729 - 2.167 | 0.410 | 1.031 | 0.596 - 1.782 | 0.914 |
| Gender |  |  |  |  |  |  |  |  |  |  |  |  |  |  |  |
| Male | Ref. |  |  | Ref. |  |  | Ref. |  |  | Ref. |  |  | Ref. |  |  |
| Female | 0.855 | 0.702 - 1.041 | 0.118 | 0.845 | 0.694 - 1.031 | 0.097 | 0.763 | 0.625 - 0.930** | 0.008 | 0.700 | 0.574 - 0.854** | <0.001 | 0.684 | 0.561 - 0.835** | <0.001 |
| Marital status |  |  |  |  |  |  |  |  |  |  |  |  |  |  |  |
| Single | Ref. |  |  | Ref. |  |  | Ref. |  |  | Ref. |  |  | Ref. |  |  |
| Married/legal partnership | 1.162 | 0.910 - 1.483 | 0.228 | 0.832 | 0.650 - 1.066 | 0.146 | 1.174 | 0.920 - 1.499 | 0.198 | 1.336 | 1.047 - 1.706* | 0.020 | 1.318 | 1.032 - 1.683* | 0.027 |
| Widowed/divorced/separated | 1.238 | 0.873 - 1.755 | 0.232 | 0.762 | 0.537 - 1.083 | 0.129 | 1.068 | 0.750 - 1.520 | 0.716 | 1.596 | 1.120 - 2.276** | 0.010 | 1.435 | 1.008 - 2.043* | 0.045 |
| *R^2^* | 0.1296 |  |  | 0.1321 |  |  | 0.1467 |  |  | 0.1312 |  |  | 0.1526 |  |  |
| N | 1494 |  |  | 1494 |  |  | 1494 |  |  | 1494 |  |  | 1494 |  |  |

* *p*<0.05; ** *p*<0.01
